# Genome‐wide association study of tuberculosis in the western Chinese Han and Tibetan population

**DOI:** 10.1002/mco2.250

**Published:** 2023-03-29

**Authors:** Hao Bai, Mengyuan Song, Shikun Lei, Lin Jiao, Xuejiao Hu, Tao Wu, Jiajia Song, Tangyuheng Liu, Wu Peng, Zhenzhen Zhao, Zirui Meng, Binwu Ying

**Affiliations:** ^1^ Department of Laboratory Medicine West China Hospital Sichuan University Chengdu P. R. China; ^2^ State Key Laboratory of Biotherapy and Cancer Center West China Hospital Sichuan University Chengdu P. R. China; ^3^ Division of Laboratory Medicine Guangdong Provincial People's Hospital Guangdong Academy of Medical Sciences Guangzhou P. R. China

**Keywords:** genome‐wide association study, human leukocyte antigen, susceptibility, tuberculosis

## Abstract

Tuberculosis (TB) remains a serious global public health threat. Accumulated evidence has demonstrated that human susceptibility to TB has a strong genetic basis. And different susceptibility single nucleotide polymorphisms (SNP) have been reported in different studies. To gain greater insight into the host susceptibility to TB, we perform a two‐stage genome‐wide association study to identify the susceptible loci of TB. In the discovery stage, 3116 (1532 TB patients and 1584 healthy controls) and 439 (211 TB patients and 228 healthy controls) individuals were genome‐wide genotyped from a western Chinese Han and Tibetan population, respectively. Based on the additive genetic model, we discovered 14 and three independent loci that had potential associations with TB susceptibility in the Chinese Han and Tibetan populations, respectively (*p* < 1 × 10^−5^). Furthermore, we conducted an imputation‐based meta‐analysis on another two East Asia cohorts to replicate our findings. We identified one independent locus harbored by the human leukocyte antigen (*HLA*) class II genes that was genome‐wide significantly associated with TB (lead SNP rs111875628 with a *p‐*value of 2.20 × 10^−9^). Our findings suggest a novel mechanism of the interaction with the *HLA* class II genes and reinforce the importance of the *HLA* class II alleles in response to TB.

## Graphical abstract

This genome‐wide association study discovered 14 and 3 independent loci that had potential associations with Tuberculosis (TB) susceptibility in the Chinese Han and Tibetan populations, respectively (*p* < 1 × 10^‐5^). Further imputation‐based meta‐analysis on another two East Asia cohorts verified one independent locus harbored by the human leukocyte antigen class II genes that was genome‐wide significantly associated with TB (lead single nucleotide polymorphism rs111875628 with a *p‐*value of 2.20 × 10^‐9^).

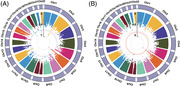

## 1 INTRODUCTION

Tuberculosis (TB), an ancient infectious disease caused by *Mycobacterium tuberculosis*, remained the leading cause of death from a single infectious agent, until the coronavirus 2019 (COVID‐19) pandemic. The World Health Organization estimated that there were up to 1.5 million individuals who died of TB in 2020, back to the level of 2017, due to reduced access to TB diagnosis and treatment during the COVID‐19 pandemic.^1^ Although about a quarter of the world's population is estimated to be infected with *Mycobacterium tuberculosis*, only less than 10% of them eventually develop active TB.^2^ Many factors including malnutrition, diabetes, HIV infection, and smoking were associated with the susceptibility to TB.^3^ In addition, accumulated evidence from historical observations, heritability estimates, linkage analyses, and genome‐wide scans has demonstrated that human susceptibility to TB has a strong genetic basis.^4^

The elucidation of host genetic differences is important to a better understanding of TB pathogenesis, prevention, and therapeutics. Previous studies have largely focused on candidate gene association studies and genome‐wide association studies (GWAS). In recent decades, candidate gene association studies have reported many well‐known susceptible variants of TB. Those variants were mainly located in immune‐related genes, including well‐studied toll‐like receptor genes, vitamin D receptor‐related genes, human leukocyte antigen (*HLA*) genes, and cytokine‐related genes.^5^, ^6^, ^7^ However, those results were often short of strong persuasion, due to the small sample size, publication bias, and possible population structure confounding.

More recently, large sample GWAS have been successfully applied to identify the host genetic susceptibility to TB. Among African and European populations, four independent GWAS have identified several susceptible variants of TB located on chromosomes 18q11.2, 11p13, 8q24, and the *HLA* class II region, respectively.^8^, ^9^, ^10^, ^11^ In addition, two GWAS in different ages of Chinese Han have been conducted.^12^, ^13^ Three SNPs harbored by *MFN2*, *RGS12*, and *HLA* class II genes were found associated with TB in a children cohort of Chinese Han.^12^ Another GWAS performed in an adult cohort of Chinese Han discovered two risk loci located on chromosomes 14q24.3 and 20p13.^13^ These findings have considerably expanded our understanding of the genetic basis of susceptibility to TB. However, those results were heterogeneous in different studies, likely because of differences in ethnic and genetic background, sample size, and inclusion criteria (TB, pulmonary TB, or *Mycobacterium tuberculosis* infection). Complex polygenic inheritance patterns are applicable for the susceptibility to TB. And these variants together accounted for only a small fraction of the risk of TB. Thus, it is vital to perform GWAS and GWAS‐based meta‐analyses for TB among the same or similar ethnic populations to further understand the hereditary basis of TB.

China has the second most estimated TB new cases in 2020, accounting for 8.5% of the global total.^1^ Especially, the incidence of TB in western China was much higher than in central and eastern China.^14^ Here, we perform a two‐stage GWAS to identify the susceptible loci of TB. In the discovery stage, 3116 (1532 TB patients and 1584 healthy controls) and 439 (211 TB patients and 228 healthy controls) individuals were genome‐wide genotyped from a western Chinese Han and Tibetan population, respectively. Based on the additive genetic model, we discovered 14 and 3 independent loci that had potential associations with TB susceptibility in the Chinese Han and Tibetan populations, respectively (*p* < 1 × 10^−5^). Furthermore, using the whole genome‐wide data from another Chinese Han population^13^ and Japanese population,^15^ we conducted an imputation‐based meta‐analysis to replicate our findings. We identified one independent locus harbored by the *HLA* class II genes that was genome‐wide significantly associated with TB (lead SNP rs111875628 with a *P* value of 2.20 × 10^−9^).

## 2 RESULTS

### 2.1 Characteristics of the discovery population

In the discovery stage, we enrolled eligible 1743 TB patients and 1812 healthy controls from a western Chinese population. First of all, we performed a principal components analysis (PCA) to investigate the population's genetic structure. They were almost clustered by two different ethnicities (Figure S1). Besides, in consideration of different geographical conditions and lifestyle habits, we analyzed the results of the Chinese Han and Tibetan populations separately. PCA analysis of the two populations showed that the cases and controls matched well (Figure S2).

The general characteristics of the discovery population based on the stratification of the age of diagnosis, sex, and clinical form are shown in Table 1. The mean age of diagnosis of Chinese Han and Tibetan TB patients was 42.47 and 34.03 years, respectively. There were more male patients than female patients, and more than 70% of patients were pulmonary TB in both populations. In addition, multiple indicators of the blood routine and biochemical test of TB patients were different from healthy individuals (Table S1). Further subgroup analysis revealed that there were also differences between Han TB patients and Tibetan TB patients in clinical characteristics (Table S2), which could be due to differences in their reference intervals of blood analysis.^16^

**TABLE 1:** Characteristics of the study population

**Characteristics**		**Han cohort (*n* = 3116)**		**Tibetan cohort (*n* = 439)**	
		**Tuberculosis (*n* = 1532)**	**Healthy control (*n* = 1584)**	**Tuberculosis (*n* = 211)**	**Healthy control (*n* = 228)**
Age of diagnosis (years) ^a^	All	42.47 ± 17.27	38.23 ± 11.28	34.03 ± 16.08	42.83 ± 12.89
	Younger (<45)	30.59 ± 8.67	32.58 ± 6.23	27.01 ± 9.62	33.09 ± 5.97
	Elder (≥45)	60.01 ± 10.54	52.83 ± 7.74	57.81 ± 9.33	55.08 ± 7.77
Sex	Male	920 (60.05%)	843 (53.22%)	111 (52.61%)	97 (42.54%)
	Female	612 (39.95%)	741 (46.78%)	100 (47.39%)	131 (57.46%)
Clinical form	Pulmonary	1202 (78.46%)	–	148 (70.14%)	–
	Extrapulmonary	330 (21.54%)	–	63 (29.86%)	–

^a^ The results were displayed as mean ± standard deviations.

### 2.2 GWAS of TB in the Chinese Han population

**FIGURE 1:**
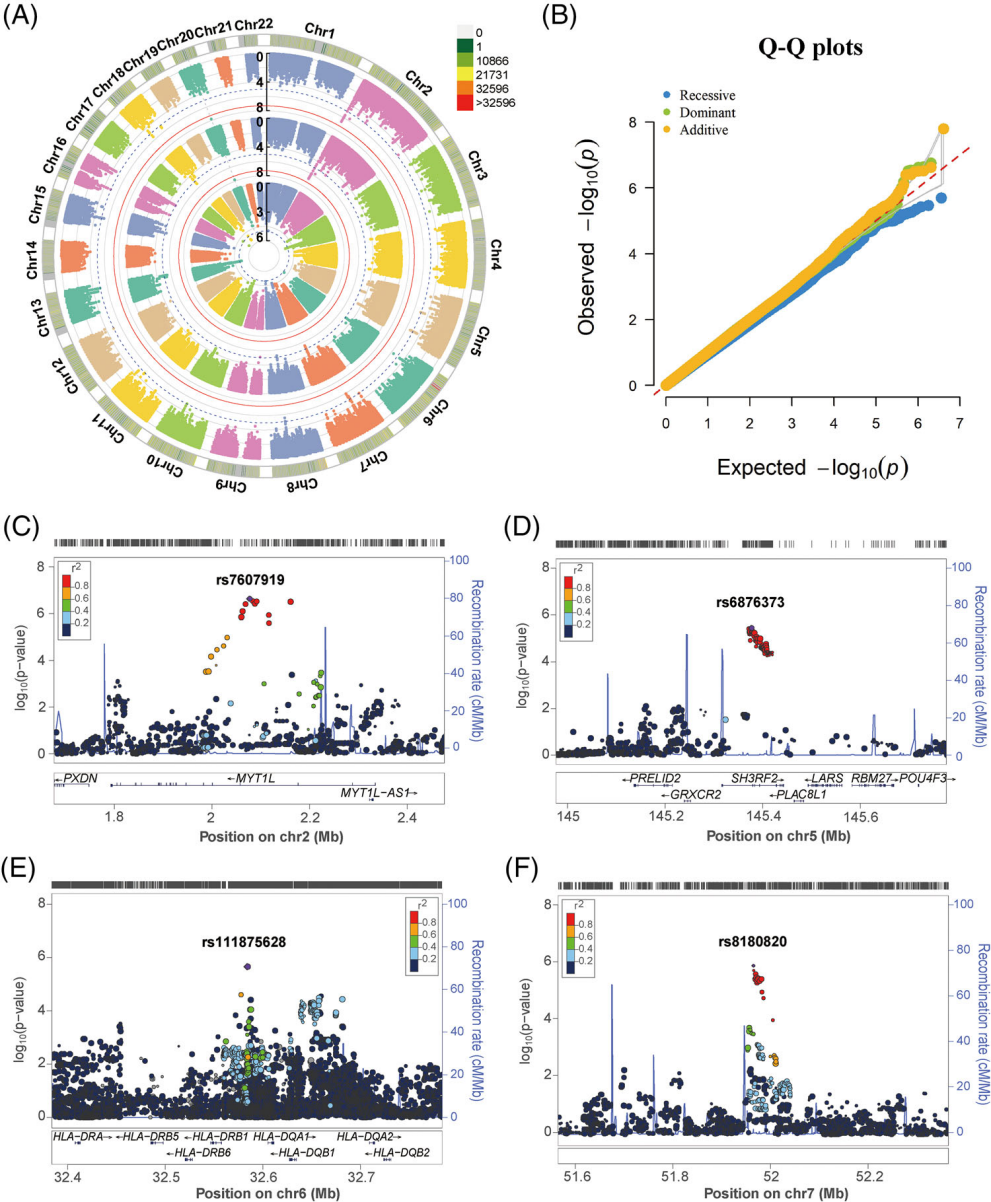
Genome‐wide association studies (GWAS) of tuberculosis (TB) in the Chinese Han population. (A) The circular Manhattan plots of the logistic regression *p‐*values under additive, dominant, and recessive models (from the outside to the inside). The dotted blue line and solid red line denote the threshold at 1 × 10^−5^ and 5 × 10^−8^, respectively. (B) The quantile‐quantile (Q‐Q) plots of the logistic regression *P* values under additive, dominant, and recessive models (λ_GC_ = 1.025, 1.021, and 0.931, respectively). (C‐F) Regional distribution of (C) rs7607919, (D) rs6876373, (E) rs111875628, and (F) rs8180820 associated with TB. The purple symbol denotes the lead single nucleotide polymorphisms (SNP), and its name is shown at the top of each plot. Recombination rates are estimated from the Asian populations of the 1000 Genomes Project (Nov 2014). Gene annotations are taken from the UCSC genome browser.

In the Chinese Han cohort, after genotype imputation and quality control, 4,096,530 SNPs were present in 1532 TB patients and 1584 healthy controls were retained. The logistic regression analysis was performed to estimate the association between SNPs and TB under three genetic models (Figure 1A). And eight significant principal components (PCs), as well as age and sex were used as covariates to correct for the population stratification. The quantile‐quantile (Q‐Q) plots are shown in Figure 1B, where the genomic inflation factors (λ_GC_) of the additive, dominant, and recessive models were 1.025, 1.021, and 0.931, respectively, indicating that effects from potential population stratification were well‐controlled.

We found 14, 11, and 4 independent loci met the significance threshold of suggestive association with TB susceptibility under additive, dominant, and recessive models, respectively (*p* < 1 × 10^−5^, Table S3 and Data S1). Among them, rs200331599 was genome‐wide significantly associated with decreased risk of TB, with a *p‐*value of 1.60 × 10^−8^ and an odds ratio (OR) of 0.103, under both additive and dominant models. However, SNP rs200331599 is a singleton and has a very low allele T frequency in the population (0.02 and 0.003 in the control and TB group, respectively). In general, it will not be considered a true association. Regional plots showed that the above‐mentioned other suggestive loci were located in the intron of *MYT1L*, *SH3RF2*, *ARHGEF28*, *NEDD4*, *LAMA3*, *DSEL‐AS1*, *PTPRD*, *NALCN*, chromosome 2p12, 2p24.1, 6p21.32 (the *HLA* class II region), 7q12.1, 7p12.3, 7q31.2, 8q22.1, 10q23.31, and 13q31.3 (Figure 1C–F and Figure S3). Among them, the *HLA* class II sequence variants have been reported to influence TB risk in populations of European and Chinese ancestry.^11^, ^12^ And the E3 ubiquitin ligase NEDD4 could enhance the killing of *Mycobacterium tuberculosis* by promoting autophagy.^17^

**FIGURE 2:**
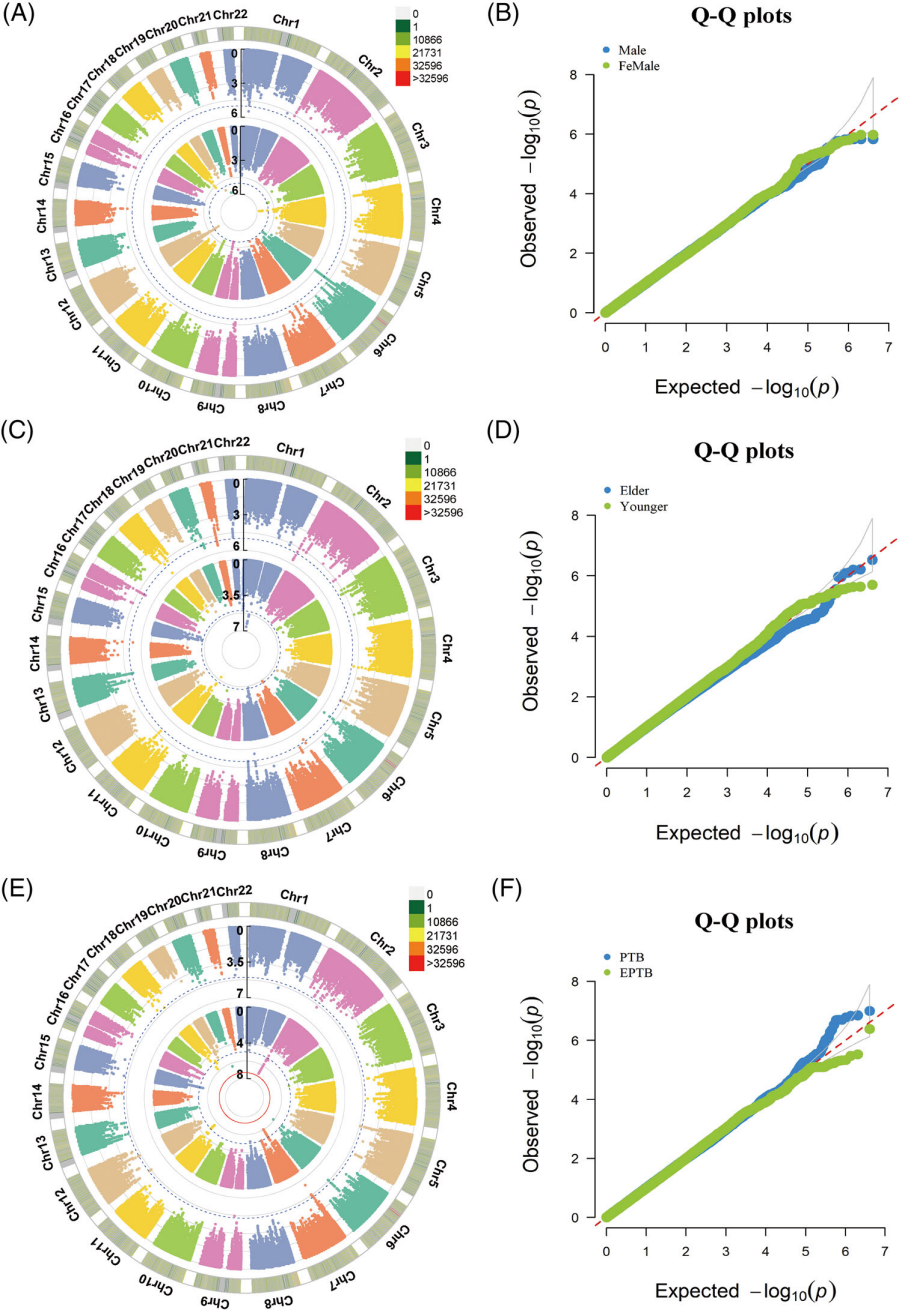
Stratified association analysis of tuberculosis (TB) in the Chinese Han population. (A) The circular Manhattan plots of the female (outside circle) and male group (inside circle) under the additive model. (B) The quantile‐quantile (Q‐Q) plots of the female and male groups under the additive model (λ_GC_ = 1.021 and 1.002, respectively). (C) The circular Manhattan plots of the younger (outside circle) and elder group (inside circle) under an additive model. (D) The Q‐Q plots of the younger and elder groups under the additive model (λ_GC_ = 1.033 and 1.015, respectively). (E) The circular Manhattan plots of extrapulmonary tuberculosis (EPTB, outside circle) and pulmonary tuberculosis group (PTB, inside circle) under an additive model. (F) The Q‐Q plots of the EPTB and PTB group under the additive model (λ_GC_ = 1.004 and 1.016, respectively). The dotted blue line in (A, C, and E) denotes the threshold at 1 × 10^−5^, and the solid red line in (E) denotes the threshold at 5 × 10^−8^.

To further minimize confounding bias and find other associations, we performed stratification analyses based on the age of diagnosis, sex, and clinical form. As shown in Figure 2 and Data S2, none of the SNPs under the additive model achieved the genome‐wide significance threshold. The association panels at the suggestive significance level were different in stratified age of diagnosis, sex, and clinical form groups. And 7/11 suggestive loci in the younger (<45 years), 5 of 5 in the elder (≥45 years), 3/4 in the male, 4/5 in the female, 1/10 in the pulmonary, and 9/9 in the extrapulmonary TB were novel and different from the results of the overall analysis (Figures S4–S6). Of note, the younger, female, and pulmonary TB group shared the same suggestive locus, rs111875628, with a *P* value of 2.33 × 10^−6^, 8.39 × 10^−6^, and 7.72 × 10^−7^, respectively (Data S2). Those results suggested that the genetic effects were different between the stratification of age, sex, and clinical form, but some common loci were observed.

### 2.3 GWAS of TB in the Chinese Tibetan population

**FIGURE 3:**
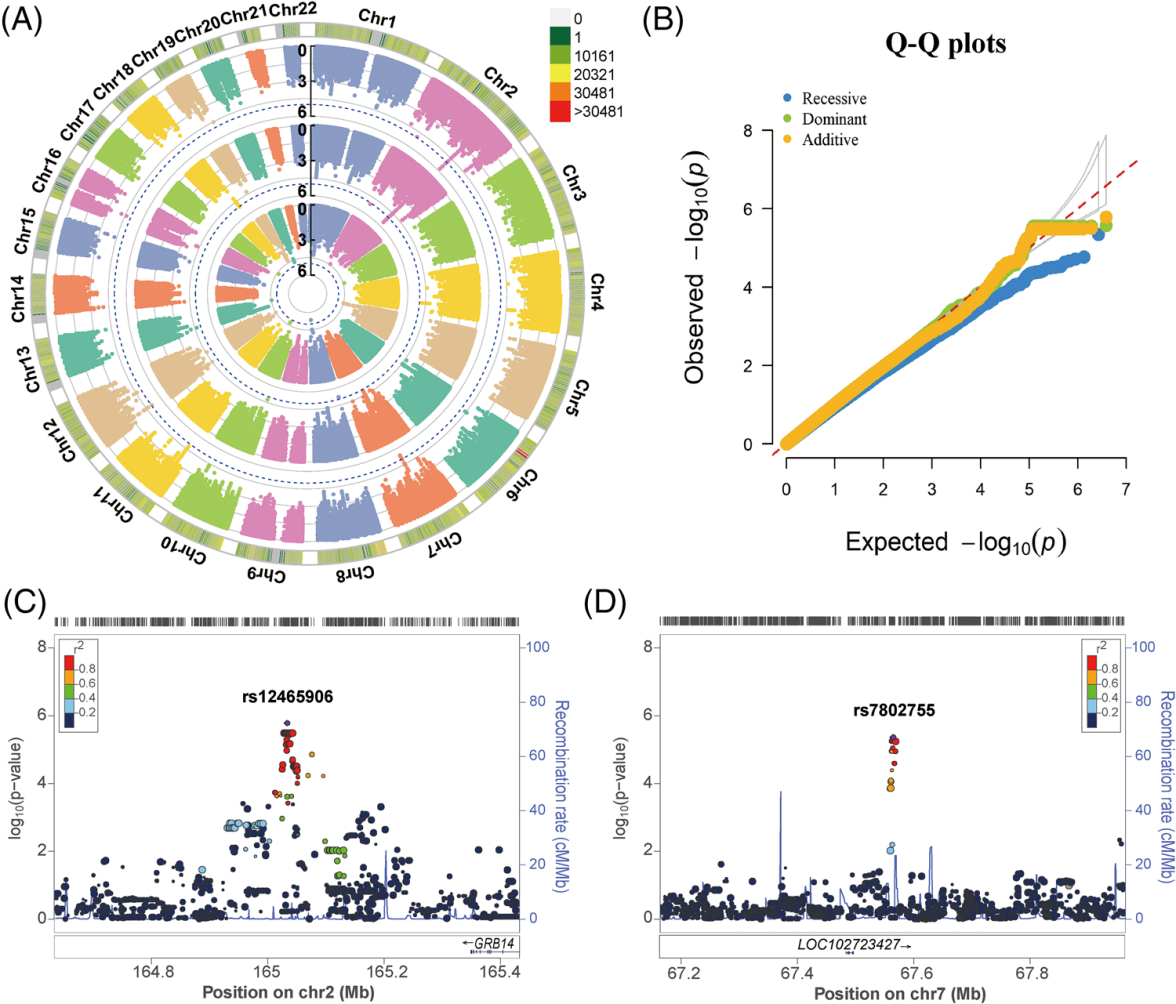
Genome‐wide association studies (GWAS) of tuberculosis (TB) in the Chinese Tibetan population. (A) The circular Manhattan plots of the logistic regression *p‐*values under additive, dominant, and recessive models (from the outside to the inside). The dotted blue line denotes the threshold at 1 × 10^−5^. (B) The quantile‐quantile (Q‐Q) plots of the logistic regression *p‐*values under additive, dominant, and recessive models (λ_GC_ = 1.018, 1.015, and 0.940, respectively). (C, D) Regional distribution of (C) rs12465906 and (D) rs7802755 associated with TB. The purple symbol denotes the lead single nucleotide polymorphisms (SNP), and its name is shown at the top of each plot. Recombination rates are estimated from the Asian populations of the 1000 Genomes Project (Nov 2014). Gene annotations are taken from the UCSC genome browser.

In the Chinese Tibetan cohort, 3,826,379 SNPs were present in 211 TB patients and 228 healthy controls were retained, after genotype imputation and quality control. Similarly, we utilized logistic regression analysis to estimate the association between SNPs and TB under additive, dominant, and recessive models (Figure 3A). After adjustment of five significant PCs, age, and sex, we did not observe significant inflation of test statistics (λ_GC_ = 1.018, 1.015, and 0.940, respectively, Figure 3B).

We found that none of the SNPs achieved the genome‐wide significance threshold in the Chinese Tibetan cohort (*p* ≤ 5 × 10^−8^). And we only found 3, 4, and 1 independent loci that met the significance threshold of suggestive association with TB susceptibility under additive, dominant, and recessive models, respectively (*p* < 1 × 10^−5^, Table S4 and Data S1). The top SNP rs12465906 on chromosome 2q24.3 was associated with a greatly increased risk of TB (*p* = 1.65 × 10^−6^, OR = 8.007, Figure 3C). SNP rs12465906 is located upstream of the *GRB14* gene, encoding the SH2 domain of growth factor receptor‐bound protein 14, which is a negative regulator of CEACAM3‐initiated bacterial phagocytosis.^18^ The second independent locus of the additive model was observed at rs7802755 downstream of the *LOC102723427* (*P* = 4.33 × 10^−6^, OR = 2.361, Figure 3D). In brief, we observed fewer SNPs in the Chinese Tibetan population than in the Han population, which was probably due to the insufficient power of less sample size.

### 2.4 Selection of candidate single nucleotide polymorphisms (SNPs) and replication in East Asia populations

**FIGURE 4:**
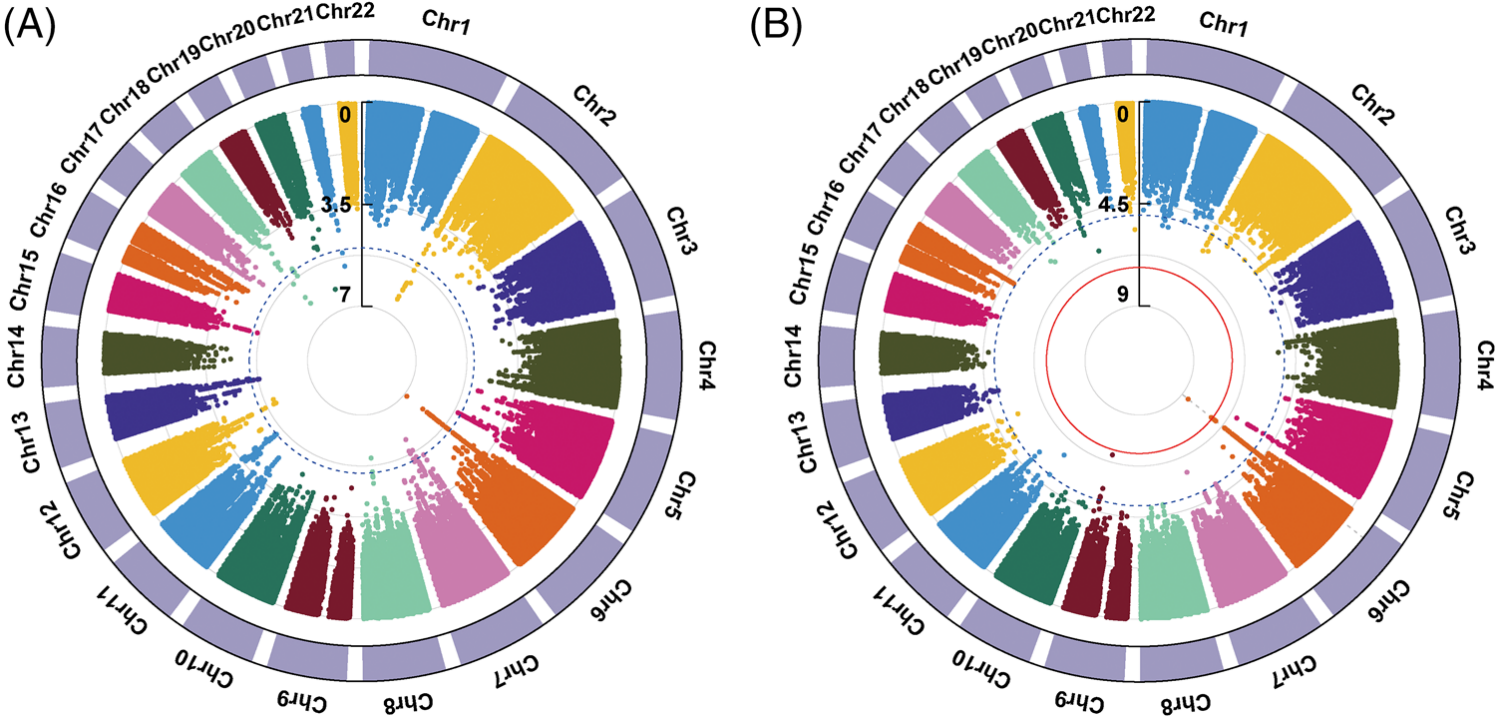
The circular Manhattan plots of meta‐analysis. The circular Manhattan plots of meta‐analysis on (A) two West China populations (discovery stage) and (B) four East Asia populations. Each dot represents a single nucleotide polymorphism (SNP). The dotted blue line and solid red line denote the threshold at 1 × 10^−5^ and 5 × 10^−8^, respectively.

Using the imputed data from the Chinese Han and Chinese Tibetan populations, we performed a genome‐wide meta‐analysis on the western Chinese populations (discovery stage, Figure 4A). We selected 12 independent SNPs with *p_*meta < 1 × 10^−5^ and the same direction of effects in the West China populations as candidate SNPs (Data S3) and replicated them in another two East Asia populations.^13^, ^15^ The suspicious SNP rs200331599 yielded opposite effects in the West China populations and was excluded from subsequent replication.

In total, the meta‐analysis on all four East Asia populations included 3125 TB cases and 214936 healthy controls. As shown in Figure 4B, we identified that two novel SNPs, rs111875628 and rs114087228, achieved the genome‐wide significance threshold (*p* = 2.20 × 10^−9^ and 4.37 × 10^−8^, respectively). Another SNP rs112925916 approached the genome‐wide significance threshold with a *p‐*value of 5.39 × 10^−8^. All of them were located in the *HLA* class II region. And the linkage disequilibrium analysis showed that they were in linkage disequilibrium with each other (Figure S7). As displayed in Table 2, the top SNP rs111875628 showed nominal significance in all four East Asia populations with a consistent direction of effect (*p* = 2.24 × 10^−6^, 1.53 × 10^−2^, 3.15 × 10^−2^, and 1.60 × 10^−2^, respectively). The allele frequencies for risk allele A were at least 0.243 in the populations. Those results indicated that the effects of rs111875628 on the susceptibility to TB were common in East Asia populations.

**TABLE 2:** Genome‐wide significant association of independent single nucleotide polymorphism (SNP) with tuberculosis (TB)

**SNP**	**Stage**	**Population**	**Samples (TB / HC)**	**MAF (TB / HC)**	**OR (95% CI)**	***p***
rs111875628 G > A Chr6:32583813 *HLA* region	Discovery	Chinese Han	1532/1584	0.293/0.243	1.337 (1.185–1.508)	2.24E‐06
		Chinese Tibetan	211/228	0.321/0.249	1.604 (1.095–2.349)	1.53E‐02
	Combined	West China	1743/1812			1.28E‐07
	Replication	Chinese Han^13^	833/1220	–	1.178 (1.029–1.327)	3.15E‐02
		Japanese^15^	549/211,904	0.443/0.411	1.161 (1.028–1.311)	1.60E‐02
	Combined	East Asia	3125/214,936			**2.20E‐09**

The chromosomal position is based on NCBI Build 37.

Abbreviations: CI, confidence interval; HC, healthy controls; MAF, minor allele frequency; OR, odds ratio; TB, tuberculosis.

### 2.5 Association of classical HLA alleles with TB

To further decipher the associations in the *HLA* region, we imputed the *HLA* region and predict classical HLA alleles for all samples of the discovery stage by SNP2HLA.^19^ Like the same as aforementioned GWAS routine analysis, the logistic regression analysis was performed to estimate the association between classical HLA alleles and TB. We found that 19 and 6 classical HLA alleles were associated with TB in the Chinese Han and Tibetan populations, respectively (*p* < 0.05, Data S4). Among them, HLA‐C*01:02, HLA‐DQA1*01:03, HLA‐DQB1*06:01, and HLA‐DQB1*04:01 showed nominal significance in both Chinese Han and Tibetan populations with a consistent direction of effect (*p* < 0.05, OR > 1). In the GWAS of the previous Chinese Han population, they also reported that several imputed classical HLA alleles including HLA‐C*01:02, HLA‐DQA1*01:03, and HLA‐DQB1*06:01 were nominally associated with TB, with the same direction of effect in the present study.^13^ Those results suggested that the HLA alleles may play important role in the predisposition to TB.

### 2.6 Performance of previously reported loci in West China populations

We also investigated the association of the previously GWAS‐identified SNPs with TB in our West China population. Among them, four SNPs (rs557011, rs9271378, rs9272785, and rs41553512) are located in the *HLA* region.^11^, ^12^ We observed a nominally significant association with TB risk in both our Chinese Han and Tibetan samples for rs557011 with a consistent direction of effect with reported results (*p* = 0.002 and 0.009, respectively). SNP rs9272785 was found to be significantly associated with TB risk in our Chinese Tibetan samples (*p* = 0.024) but failed to replicate the association in our Chinese Han population. Besides, we observed the same protective effect for rs2057178^9^ mutation in our Chinese Han population (*p* = 0.029). But we failed to replicate the association of rs41553512, rs2269497, rs4240897,^12^ rs9271378,^11^ rs4331426,^8^ rs10956514, and rs4733781^10^ in our Chinese population (Table S5), which might be due to the differences of genetic background, mutation frequency, sample size, and inclusion criteria in different studies.

### 2.7 Quantitative trait loci and susceptibility gene analysis

**FIGURE 5:**
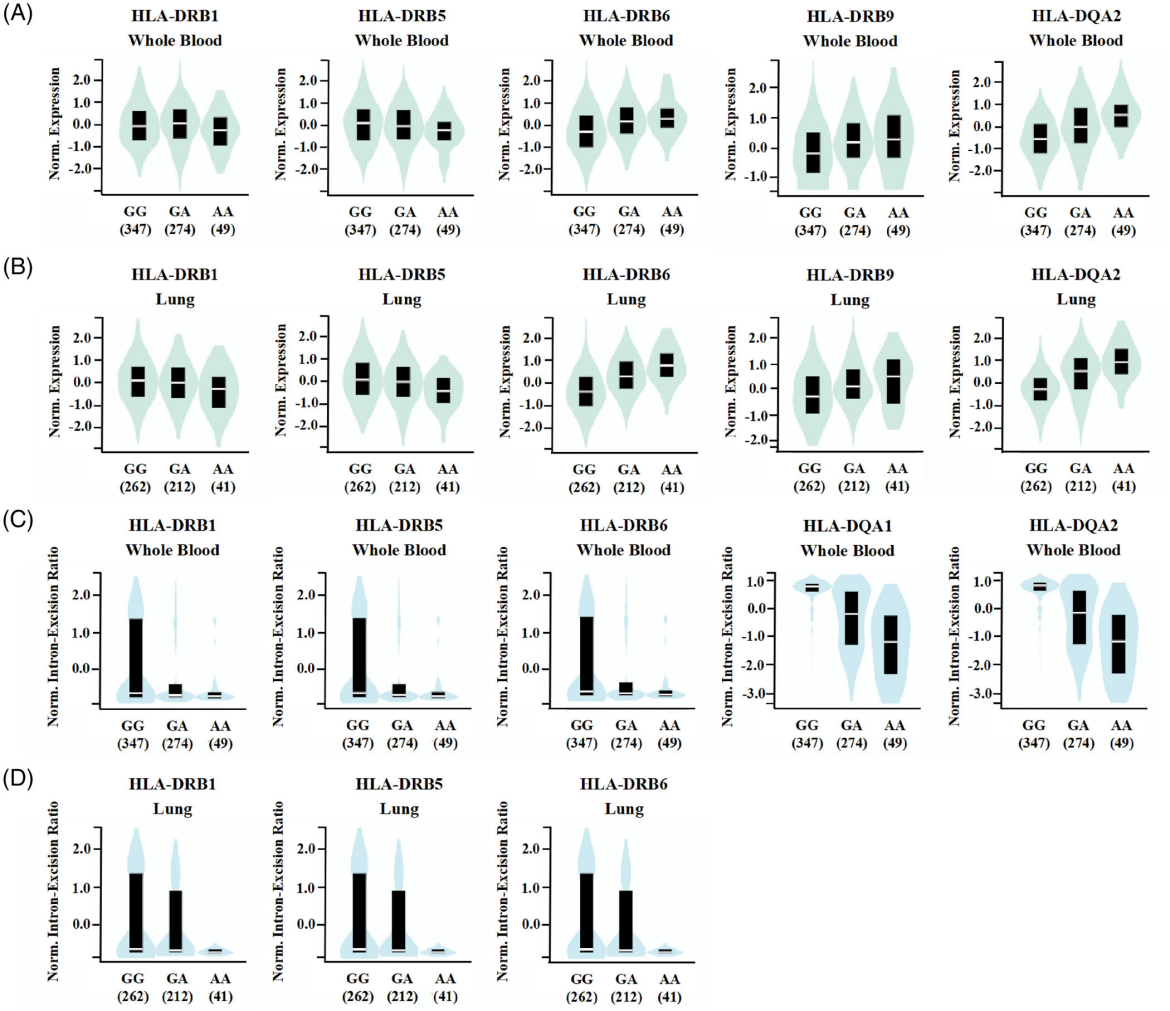
The expression quantitative trait loci (eQTL) and sQTL effects of rs111875628 on the *HLA* class II genes. (A, B) The eQTL effects of rs111875628 on the *HLA* class II genes in (A) the whole blood and (B) lung tissues. (C, D) The splicing quantitative trait loci (sQTL) effects of rs111875628 on the *HLA* class II genes in (C) the whole blood and (D) lung tissues. The data were downloaded from the GTEx portal.

Functional variants may play important roles in disease phenotypes by regulating gene expression levels or splicing modes of introns. To predict the effects of variants on gene expression and splicing, we analyzed all independent loci with *P* values of < 1 × 10^−5^ in our Chinese Han and Tibetan populations using the GTEx portal. In total, we discovered that 12 suggestive loci had either the expression quantitative trait loci (eQTL) or the splicing quantitative trait loci (sQTL) (Table S6). Especially, the results showed that the rs111875628 significantly influence both the expression and splicing of the *HLA* class II genes in multiple tissues (169 eQTL hits and 107 sQTL hits, as displayed in Data S5). The A allele carriers of rs111875628 with a higher risk for TB had significantly higher *HLA‐DQA2*, *HLA‐DRB6*, and *HLA‐DRB9* expression, and lower *HLA‐DRB1* and *HLA‐DRB5* expression in both whole blood and lung tissues (Figure 5A,B). However, further colocalization analysis revealed that the expressions of those genes and TB susceptibility were not associated with the same causal variant (Table S7). And the A allele carriers of rs111875628 also had a significantly lower intron‐excision ratio of *HLA‐DQA1* and *HLA‐DQA2* in the whole blood, and *HLA‐DRB1*, *HLA‐DRB5*, and *HLA‐DRB6* genes in both whole blood and lung tissues (Figure 5C,D). Based on the above results, the mechanism by which rs111875628 participates in TB susceptibility still requires clarification.

To identify genes associated with TB susceptibility, we used MAGMA software to perform gene‐based tests that combine the SNP associations into genic annotations. The results showed that no gene met the genome‐wide significance after Bonferroni correction in either the Chinese Han or Chinese Tibetan population. The top five genes associated with TB susceptibility in the Chinese Han and Tibetan population were *SH3RF2*, *LIPA*, *SNTG1*, *RP9*, and *IFIT1B*, and *RERGL*, *HAS2*, *HOXC4*, *FAM177B*, and *TNNT1*, respectively (Figure S8). The gene‐set enrichment analysis identified that the “GO: negative regulation of high voltage‐gated calcium channel (VGCC) activity” is significant for TB susceptibility in the Chinese Han population (*p* = 0.014 after Bonferroni correction, Table S8). Previous research has revealed that VGCC plays a negative role in *Mycobacterium tuberculosis* infection by regulating calcium mobilization in cells that determine protective immunity.^20^ While none of the gene sets is significant for TB susceptibility in the Chinese Tibetan population (Table S9).

## 3 DISCUSSION

Complex polygenic inheritance patterns are applicable for the susceptibility to TB. Although previous GWAS have identified several genetic variants associated with TB susceptibility, those results were heterogeneous in different populations. Thus, it is still desirable to perform GWAS and GWAS‐based meta‐analyses for TB among the same or similar ethnic groups to further understand the genetic basis of TB. In the present study, we discovered several independent loci that had potential associations with TB susceptibility in the Chinese Han and Tibetan populations, respectively. And we identified one novel locus in the *HLA* region that was significantly associated with TB risk in the East Asia populations, by genome‐wide imputation‐based meta‐analysis. We further demonstrated that the rs111875628 may influence the splicing of the *HLA* class II genes in multiple tissues. These genes have key roles in immune responses as discussed below.

The HLA is the most polymorphic biological system with codominant expression, which is central to physiology, protective immunity, and deleterious, disease‐causing autoimmune reactivity.^21^ The *HLA* class I (*HLA‐A*, *HLA‐B*, and *HLA‐C*) and *HLA* class II (*HLA‐DR*, *HLA‐DQ*, and *HLA‐DP*) genes encode classical major histocompatibility complex (MHC) class I and II molecules identified for their role in the presentation of antigen to CD8^+^ and CD4^+^ T cells, respectively.^22^ Researchers have discovered that the *HLA* variations are associated with the occurrence and development of multiple diseases, particularly infections, and autoimmune diseases.^23^, ^24^ Previous candidate gene studies of TB susceptibility have focused on the *HLA* class II genes and yielded conflicting results. Further pooled meta‐analysis of cross‐population demonstrated that polymorphisms in *HLA‐DRB1*, *HLA‐DQB1*, *HLA‐DQA1*, and *HLA‐DRB5* genes are associated with TB.^25^, ^26^

Using the genome‐wide imputation data of four East Asia populations, we identified an independent locus (lead SNP rs111875628) in the *HLA* class II region that was significantly associated with an increased risk of TB. This is the third independent line of GWAS evidence showing the robust association of *HLA* class II with TB to date.^11^, ^12^ The first evidence of GWAS reported three SNPs (rs557011, rs9271378, and rs9272785) in the *HLA* class II region in Europeans.^11^ For the top SNP rs557011, a nominally significant association with a consistent direction of effect was also observed in our western Chinese Han, Chinese Tibetan (Table S5), and other independent Chinese Han samples.^13^ The second evidence of GWAS reported a missense variant of *HLA‐DRB5*, rs41553512, was significantly associated with TB and was classified as damaging in a children cohort of Han Chinese.^12^ However, we failed to replicate the association of rs41553512 in our data set (Table S5), which might be due to the mutation rarity (MAF = 0.023 and 0.004 in our Chinese Han and Tibetan samples, respectively) as well as the distinct genetic predisposition to TB in children and adults.^27^

As a complex infectious disease, TB is a consequence of the intricate interaction between polygenic inheritance and environmental factors. The newly identified TB‐associated loci in the East Asia populations (top three SNPs: rs111875628, rs114087228, and rs112925916) are also common in other populations (MAF: 0.182–0.476, Table S10), but were not reported previously. And our data, in turn, replicated only part of the previously reported susceptibility loci to TB (Table S5). Such inconsistency in GWAS across cohorts from different populations may be due to differences in the genetic background (e.g., different causal variants and linkage disequilibrium structures) and environmental factors (e.g., malnutrition and smoking) as well as differences in study design (e.g., SNP arrays, sample size, and inclusion criteria).^28^, ^29^

The present study provided novel insights into the genetic basis of TB in the western Chinese Han and Tibetan populations. However, our study had some limitations. In the present study, we recruited healthy controls from the same geographical area of the TB cases to reduce the confounding environmental factors as far as possible. And we corrected for population stratification, as well as the influence of age and sex. Despite that, more non‐genetic contributors to TB including the measurement of *Mycobacterium tuberculosis* sublineages and infection status of controls are lacking. Although we made the first attempt to identify TB‐related SNPs in the Tibetan population, the sample size of the Tibetan cohort is not large enough to discover genome‐wide significant loci. In addition to the small sample size, the differences in terms of genetics, geographical conditions, and lifestyle habits may also contribute to the heterogeneity between Han and Tibetan cohorts. Due to the different genetic coverage in the four East Asia cohorts, the lack of some SNPs in certain cohorts reduced the test power of meta‐analysis. And additional studies will be required to validate our results.

Taken together, our study revealed a number of novel potential risk loci of TB in the western Chinese Han and Tibetan populations. And we identified one independent locus (lead SNP rs111875628) in the *HLA* class II region that was significantly associated with TB risk in the East Asia populations. These results provide additional clues to understanding the host genetic contribution to TB susceptibility and reinforce the importance of the *HLA* class II alleles in response to TB.

## 4 METHODS

### 4.1 Samples

Participants in the present case‐control study were enrolled in western China. All TB patients were confirmed by two experienced physicians, based on clinical symptoms, bacteriological or pathological evidence (one of smear microscopy, culture, or TB‐DNA positive), radiological findings of active TB, and appropriate responses to anti‐TB therapy. Healthy controls were recruited from the same geographical area as the TB cases to reduce the confounding environmental factors as far as possible. All healthy controls were asymptomatic, with normal erythrocyte sedimentation rate, C‐reactive protein level, and chest X‐ray results, and without a history of TB. *Mycobacterium tuberculosis* infection status of these controls was unknown. Individuals with chronic use of corticosteroids, immunodeficiency, HIV infection, or other infectious diseases were excluded. Samples of the Chinese Han cohort were recruited from the West China Hospital of Sichuan University and The Public Health Clinical Center of Chengdu. After quality control, a total of 1532 TB patients and 1584 healthy controls remained. And 211 TB patients and 228 healthy controls collected from the Tibet Autonomous Region People's Government Chengdu Office Hospital were ultimately analyzed in the Tibetan cohort.

The clinical features and laboratory test results of all participants were collected from their electronic records. The differences in laboratory indexes between the two groups were tested by the independent samples t‐test and the Mann‐Whitney U test according to the normality of data using SPSS version 22.0 (IBM, USA). Statistical significance was set at *p* < 0.05.

### 4.2 Genomic DNA extraction and genotyping

We collected a tube of peripheral venous blood from each participant. Genomic DNA was extracted using QIAamp DNA Blood Mini Kits (Qiagen, Germany) according to the manufacturer's instructions. All DNA samples were quantified using a NanoDrop ND‐1000 spectrophotometer (Thermo, USA) and agarose gel electrophoresis. The extracted DNA was diluted to working concentrations of 50 ng/µL and was genotyped by Genergy Bio‐Technology (Shanghai, China) using the HumanOmniExpress BeadChip (Illumina, USA) following the manufacturer's specifications.

### 4.3 Quality control and genotype imputation

To obtain high‐quality data for the GWAS, we pruned the genome‐wide genotyping data using Plink.^30^ First, SNPs with call rates < 95%, minor allele frequency (MAF) < 1%, located on the Y chromosome, or significant deviations from the Hardy‐Weinberg equilibrium (*p* ≤ 1 × 10^−5^) in the control group were excluded. Second, samples with a call rate < 95%, identity by descent (IBD) > 0.1875, or ambiguous sex (0.2 < F value of sex check < 0.8) were excluded. Third, we used the Python snpflip package (https://pypi.org/project/snpflip/) to check reverse and ambiguous strand SNPs. Then the reverse strand SNPs were flipped to the forward strand and ambiguous strand SNPs were removed by Plink software. To maximize genetic coverage, we used the SHAPEIT software^31^ to pre‐phase the haplotypes in each chromosome. And ungenotyped SNPs were imputed using IMPUTE2 software^32^ based on a reference panel from the 1000 Genomes Project phase I integrated variant set (version 3, b37, Mar 2012). The imputed variants with INFO ≥ 0.8 remained for further analysis. And then imputed SNPs with call rates < 95%, MAF < 1%, or significant deviations from the Hardy‐Weinberg equilibrium (*p* ≤ 1 × 10^−5^) in the control group were excluded using Plink.

### 4.4 Association analysis

First, we performed a PCA using Plink. As different populations have different degrees of population stratification, we then used the stats method of EIGENSTRAT software^33^ to test the significance of the top 20 PCs. The logistic regression analysis was performed to estimate the association between SNPs and TB under additive, dominant, and recessive models. And significant PCs (*p* < 0.05), together with the age of diagnosis and sex, were used as covariates in the logistic regression analysis to correct for the population stratification. To further minimize confounding bias and find other associations, we performed stratification analyses based on the age of diagnosis, sex, and clinical form. The cutoff age of diagnosis was set at 45 years, for the genetic effects are expected to be stronger in young patients than in older ones.^34^, ^35^ The Manhattan plots and Q‐Q plots of this test were constructed using the CMplot package of R.^36^ Regional association and linkage disequilibrium were generated using the online tool LocusZoom.^37^ The threshold for genome‐wide significance was set at *p* < 5 × 10^−8^. And the significance threshold of suggestive association was set at *p* < 1 × 10^−5^. Furthermore, conditional logistic regression analyses were applied to evaluate the independent effects of suggestive SNPs.

### 4.5 Imputation and association analysis of classical HLA alleles

First, SNPs located in chr6: 25−35 MB were extracted by Plink. Then we used SNP2HLA^19^ to impute the ungenotyped variants and predict the classical HLA alleles with the Pan‐Asian reference panel.^38^ After imputation, 8245 SNPs were presented. Also, the logistic regression analysis was performed to estimate the associations using significant PCs, as well as age and sex as covariates.

### 4.6 Replication by imputation‐based meta‐analysis

We obtained the imputed genome‐wide data of another Chinese Han (833 TB patients and 1220 healthy controls)^13^ and a Japanese population (549 TB patients and 211,904 controls).^15^ With obtained data, we performed an imputation‐based meta‐analysis of TB in East Asia populations (Chinese and Japanese) using METAL^39^ with the following parameters: EFFECT, log (OR); STDERR, standard error; Weights in *p*‐value Based Analysis, sample size. As shown in Table S11, power estimates for the total sample size used in the current study (3125 cases and 214936 controls) were calculated with the GAS Power Calculator,^40^ giving a population incidence of approximately 0.0002,^1^ and a significance level of 5 × 10^−8^.

### 4.7 Functional annotation

We queried all suggestive association loci to the GTEx portal^41^ and obtained all eQTL and sQTL of them. Besides, we conducted a colocalization analysis to determine if the same variants were responsible for the TB association signal and the eQTL signal by R package Coloc.^42^ We extracted all SNPs residing within chr6: 25–35 MB from the summary association statistics and public eQTL dataset of blood and lung (GTEx). Colocalization was defined as the posterior probability of H4 (PP_H4_) greater than 0.80. Gene‐based and gene‐set enrichment analyses were performed using the FUMA pipeline.^43^ Input SNPs of the Chinese Han and Tibetan populations were mapped to 17891 and 17733 protein‐coding genes, respectively. The significance was defined at *p* = 1 × 10^−5^. Using the result of gene analysis, gene‐set analysis is performed with default parameters using MAGMA v1.06.^44^ MAGMA gene‐set analysis is performed for curated gene sets and GO terms obtained from MsigDB.^45^, ^46^

## AUTHOR CONTRIBUTIONS

Hao Bai, Xuejiao Hu, Tao Wu, Jiajia Song, Tangyuheng Liu, Wu Peng, Zhenzhen Zhao, and Zirui Meng collected samples and conducted the experiments. Hao Bai, Mengyuan Song, Shikun Lei, and Lin Jiao conducted the data analysis. Hao Bai wrote the manuscript. Binwu Ying designed the study and revised the article. All authors read and approved the final manuscript.

## CONFLICT OF INTEREST STATEMENT

The authors declare no conflict of interest.

## ETHICS STATEMENT

This study was approved by the Ethics Committee of West China Hospital of Sichuan University (permit number: 2019−829) and was conducted according to the Declaration of Helsinki principles. Signed informed consent was obtained from each participant.

## Supporting information



Supporting information

Supporting information

Supporting information

Supporting information

Supporting information

Supporting information

## Data Availability

All data are available from the corresponding authors upon request. The data are not publicly available due to ethical restrictions.

## References

[1] Geneva: World Health Organization . Global tuberculosis report 2021. 2021. License: CC BY‐NC‐SA 3.0 IGO.

[2] Zumla A , Raviglione M , Hafner R , von Reyn CF . Tuberculosis. N Engl J Med. 2013;368(8):745‐755. 23425167 10.1056/NEJMra1200894

[3] Dye C , Lonnroth K , Jaramillo E , Williams BG , Raviglione M . Trends in tuberculosis incidence and their determinants in 134 countries. Bull World Health Organ. 2009;87(9):683‐691. 19784448 10.2471/BLT.08.058453 PMC2739916

[4] Abel L , Fellay J , Haas DW , et al. Genetics of human susceptibility to active and latent tuberculosis: present knowledge and future perspectives. Lancet Infect Dis. 2018;18(3):e64‐e75. 29111156 10.1016/S1473-3099(17)30623-0 PMC8903186

[5] Naranbhai V . The role of host genetics (and genomics) in tuberculosis. Microbiol Spectr. 2016;4(5). 10.1128/microbiolspec.TBTB2-0011-2016 27787193

[6] Kinnear C , Hoal EG , Schurz H , van Helden PD , Moller M . The role of human host genetics in tuberculosis resistance. Expert Rev Respir Med. 2017;11(9):721‐737. 28703045 10.1080/17476348.2017.1354700

[7] van Tong H , Velavan TP , Thye T , Meyer CG . Human genetic factors in tuberculosis: an update. Trop Med Int Health. 2017;22(9):1063‐1071. 28685916 10.1111/tmi.12923

[8] Thye T , Vannberg FO , Wong SH , et al. Genome‐wide association analyses identifies a susceptibility locus for tuberculosis on chromosome 18q11.2. Nat Genet. 2010;42(9):739‐741. 20694014 10.1038/ng.639 PMC4975513

[9] Thye T , Owusu‐Dabo E , Vannberg FO , et al. Common variants at 11p13 are associated with susceptibility to tuberculosis. Nat Genet. 2012;44(3):257‐259. 22306650 10.1038/ng.1080 PMC3427019

[10] Curtis J , Luo Y , Zenner HL , et al. Susceptibility to tuberculosis is associated with variants in the ASAP1 gene encoding a regulator of dendritic cell migration. Nat Genet. 2015;47(5):523‐527. 25774636 10.1038/ng.3248 PMC4414475

[11] Sveinbjornsson G , Gudbjartsson DF , Halldorsson BV , et al. HLA class II sequence variants influence tuberculosis risk in populations of European ancestry. Nat Genet. 2016;48(3):318‐322. 26829749 10.1038/ng.3498 PMC5081101

[12] Qi H , Zhang YB , Sun L , et al. Discovery of susceptibility loci associated with tuberculosis in Han Chinese. Hum Mol Genet. 2017;26(23):4752‐4763. 29036319 10.1093/hmg/ddx365

[13] Zheng R , Li Z , He F , et al. Genome‐wide association study identifies two risk loci for tuberculosis in Han Chinese. Nat Commun. 2018;9(1):4072. 30287856 10.1038/s41467-018-06539-w PMC6172286

[14] Gao L , Lu W , Bai L , et al. Latent tuberculosis infection in rural China: baseline results of a population‐based, multicentre, prospective cohort study. Lancet Infect Dis. 2015;15(3):310‐319. 25681063 10.1016/S1473-3099(14)71085-0

[15] Ishigaki K , Akiyama M , Kanai M , et al. Large‐scale genome‐wide association study in a Japanese population identifies novel susceptibility loci across different diseases. Nat Genet. 2020;52(7):669‐679. 32514122 10.1038/s41588-020-0640-3 PMC7968075

[16] Yang W , Zhao S , Liu D , Su G , Guan XJHAM . Establishment of reference intervals for blood cell analysis of Adult Tibetan farmers and herdsmen over 4100 meters above sea level in Tibet based on a health survey. Biology. 2020;21(3). 10.1089/ham.2020.0006 32498572

[17] Pei G , Buijze H , Liu H , et al. The E3 ubiquitin ligase NEDD4 enhances killing of membrane‐perturbing intracellular bacteria by promoting autophagy. Autophagy. 2017;13(12):2041‐2055. 29251248 10.1080/15548627.2017.1376160 PMC5788543

[18] Kopp K , Buntru A , Pils S , et al. Grb14 is a negative regulator of CEACAM3‐mediated phagocytosis of pathogenic bacteria. J Biol Chem. 2012;287(46):39158‐39170. 22948154 10.1074/jbc.M112.395228 PMC3493956

[19] Jia X , Han B , Onengut‐Gumuscu S , et al. Imputing amino acid polymorphisms in human leukocyte antigens. PLoS One. 2013;8(6):e64683. 23762245 10.1371/journal.pone.0064683 PMC3675122

[20] Gupta S , Salam N , Srivastava V , et al. Voltage gated calcium channels negatively regulate protective immunity to Mycobacterium tuberculosis. PLoS One. 2009;4(4):e5305. 19390594 10.1371/journal.pone.0005305 PMC2669286

[21] Dendrou CA , Petersen J , Rossjohn J , Fugger L . HLA variation and disease. Nat Rev Immunol. 2018;18(5):325‐339. 29292391 10.1038/nri.2017.143

[22] Blum JS , Wearsch PA , Cresswell P . Pathways of antigen processing. Annu Rev Immunol. 2013;31:443‐473. 23298205 10.1146/annurev-immunol-032712-095910 PMC4026165

[23] Tian C , Hromatka BS , Kiefer AK , et al. Genome‐wide association and HLA region fine‐mapping studies identify susceptibility loci for multiple common infections. Nat Commun. 2017;8(1):599. 28928442 10.1038/s41467-017-00257-5 PMC5605711

[24] Sakaue S , Kanai M , Tanigawa Y , et al. A cross‐population atlas of genetic associations for 220 human phenotypes. Nat Genet. 2021;53(10):1415‐1424. 34594039 10.1038/s41588-021-00931-x PMC12208603

[25] Oliveira‐Cortez A , Melo AC , Chaves VE , Condino‐Neto A , Camargos P . Do HLA class II genes protect against pulmonary tuberculosis? A systematic review and meta‐analysis. Eur J Clin Microbiol Infect Dis. 2016;35(10):1567‐1580. 27412154 10.1007/s10096-016-2713-x

[26] Jiao L , Song J , Chen H , et al. Genetic architecture of tuberculosis susceptibility: a comprehensive research synopsis, meta‐analyses, and epidemiological evidence. Infect Genet Evol. 2022;104:105352. 35998870 10.1016/j.meegid.2022.105352

[27] Alcais A , Fieschi C , Abel L , Casanova JL . Tuberculosis in children and adults: two distinct genetic diseases. J Exp Med. 2005;202(12):1617‐1621. 16365144 10.1084/jem.20052302 PMC2212964

[28] Tam V , Patel N , Turcotte M , Bosse Y , Pare G , Meyre D . Benefits and limitations of genome‐wide association studies. Nat Rev Genet. 2019;20(8):467‐484. 31068683 10.1038/s41576-019-0127-1

[29] Peterson RE , Kuchenbaecker K , Walters RK , et al. Genome‐wide association studies in ancestrally diverse populations: opportunities, methods, pitfalls, and recommendations. Cell. 2019;179(3):589‐603. 31607513 10.1016/j.cell.2019.08.051 PMC6939869

[30] Purcell S , Neale B , Todd‐Brown K , et al. PLINK: a tool set for whole‐genome association and population‐based linkage analyses. Am J Hum Genet. 2007;81(3):559‐575. 17701901 10.1086/519795 PMC1950838

[31] Delaneau O , Zagury JF , Marchini J . Improved whole‐chromosome phasing for disease and population genetic studies. Nat Methods. 2013;10(1):5‐6. 23269371 10.1038/nmeth.2307

[32] Howie B , Fuchsberger C , Stephens M , Marchini J , Abecasis GR . Fast and accurate genotype imputation in genome‐wide association studies through pre‐phasing. Nat Genet. 2012;44(8):955‐959. 22820512 10.1038/ng.2354 PMC3696580

[33] Price AL , Patterson NJ , Plenge RM , Weinblatt ME , Shadick NA , Reich D . Principal components analysis corrects for stratification in genome‐wide association studies. Nat Genet. 2006;38(8):904‐909. 16862161 10.1038/ng1847

[34] Jiang X , Holmes C , McVean G . The impact of age on genetic risk for common diseases. PLoS Genet. 2021;17(8):e1009723. 34437535 10.1371/journal.pgen.1009723 PMC8389405

[35] Mahasirimongkol S , Yanai H , Mushiroda T , et al. Genome‐wide association studies of tuberculosis in Asians identify distinct at‐risk locus for young tuberculosis. J Hum Genet. 2012;57(6):363‐367. 22551897 10.1038/jhg.2012.35

[36] Yin L , Zhang H , Tang Z , et al. rMVP: a memory‐efficient, visualization‐enhanced, and parallel‐accelerated tool for genome‐wide association study. Genom Proteom Bioinformatics. 2021;19(4):619‐628. 10.1016/j.gpb.2020.10.007 PMC9040015 33662620

[37] Pruim RJ , Welch RP , Sanna S , et al. LocusZoom: regional visualization of genome‐wide association scan results. Bioinformatics. 2010;26(18):2336‐2337. 20634204 10.1093/bioinformatics/btq419 PMC2935401

[38] Pillai NE , Okada Y , Saw WY , et al. Predicting HLA alleles from high‐resolution SNP data in three Southeast Asian populations. Hum Mol Genet. 2014;23(16):4443‐4451. 24698974 10.1093/hmg/ddu149

[39] Willer CJ , Li Y , Abecasis GR . METAL: fast and efficient meta‐analysis of genomewide association scans. Bioinformatics. 2010;26(17):2190‐2191. 20616382 10.1093/bioinformatics/btq340 PMC2922887

[40] Skol AD , Scott LJ , Abecasis GR , Boehnke M . Joint analysis is more efficient than replication‐based analysis for two‐stage genome‐wide association studies. Nat Genet. 2006;38(2):209‐213. 16415888 10.1038/ng1706

[41] Consortium GT . The genotype‐tissue expression (GTEx) project. Nat Genet. 2013;45(6):580‐585. 23715323 10.1038/ng.2653 PMC4010069

[42] Giambartolomei C , Vukcevic D , Schadt EE , et al. Bayesian test for colocalisation between pairs of genetic association studies using summary statistics. PLoS Genet. 2014;10(5):e1004383. 24830394 10.1371/journal.pgen.1004383 PMC4022491

[43] Watanabe K , Taskesen E , van Bochoven A , Posthuma D . Functional mapping and annotation of genetic associations with FUMA. Nat Commun. 2017;8(1):1826. 29184056 10.1038/s41467-017-01261-5 PMC5705698

[44] de Leeuw CA , Mooij JM , Heskes T , Posthuma D . MAGMA: generalized gene‐set analysis of GWAS data. PLoS Comput Biol. 2015;11(4):e1004219. 25885710 10.1371/journal.pcbi.1004219 PMC4401657

[45] Subramanian A , Tamayo P , Mootha VK , et al. Gene set enrichment analysis: a knowledge‐based approach for interpreting genome‐wide expression profiles. Proc Nat Acad Sci. 2005;102(43):15545‐15550. 16199517 10.1073/pnas.0506580102 PMC1239896

[46] Liberzon A , Birger C , Thorvaldsdottir H , Ghandi M , Mesirov JP , Tamayo P . The Molecular Signatures Database (MSigDB) hallmark gene set collection. Cell Syst. 2015;1(6):417‐425. 26771021 10.1016/j.cels.2015.12.004 PMC4707969

