# Supplementary material for: Genome‐wide association study of tuberculosis in the western Chinese Han and Tibetan population
Source: MedComm (2020). 2023 Mar 29;4(2):e250. doi: 10.1002/mco2.250 (PMC10050958; doi:10.1002/mco2.250)
Supplement: Supplementary file 1 — Supporting information [file MCO2-4-e250-s001.docx]

**Supplementary Information**

**Genome-wide association study of tuberculosis in the western Chinese Han and Tibetan population**

**Authors:** Hao Bai^1#^, Mengyuan Song^1#^, Shikun Lei^2#^, Lin Jiao^1^, Xuejiao Hu^3^, Tao Wu^1^, Jiajia Song^1^, Tangyuheng Liu^1^, Wu Peng^1^, Zhenzhen Zhao^1^, Zirui Meng^1^, Binwu Ying^1^*.

**Affiliations:**

^1^ Department of Laboratory Medicine, West China Hospital, Sichuan University, Chengdu, Sichuan, P. R China;

^2^ State Key Laboratory of Biotherapy and Cancer Center, West China Hospital, Sichuan University, Chengdu, Sichuan, P. R China;

^3^ Division of Laboratory Medicine, Guangdong Provincial People's Hospital, Guangdong Academy of Medical Sciences, Guangzhou, Guangdong, P. R China;

^#^ These authors contributed equally to this work.

* Corresponding author: Binwu Ying; Email: yingbinwu@scu.edu.cn; Address: No. 37 Guoxue Lane, West China Hospital, Sichuan University, Chengdu, Sichuan, P.R China; Tel: 86-28-85422533; Fax: 86-28-85422533.

**Table S1. Laboratory indexes of the study population.**

| **Laboratory indexes** | **Tuberculosis**  **(n = 1743)** | **Healthy control**  **(n = 1812)** | ***P*** |
| --- | --- | --- | --- |
| Hematocrit ^a^ (%) | 0.38 ± 0.07 | 0.43 ± 0.04 | < 0.001 |
| Hemoglobin ^a^ (g/L) | 124.41 ± 23.42 | 144.46 ± 15.33 | < 0.001 |
| Erythrocyte ^a^ (×10^12^/L) | 4.36 ± 0.76 | 4.82 ± 0.50 | < 0.001 |
| Platelet ^a^ (×10^9^/L) | 257.68 ± 129.34 | 198.77 ± 53.63 | < 0.001 |
| Leukocyte ^a^ (×10^9^/L) | 7.34 ± 3.37 | 5.90 ± 1.49 | < 0.001 |
| Monocyte ^a^ (×10^9^/L) | 0.50 ± 0.28 | 0.33 ± 0.13 | < 0.001 |
| Albumin ^a^ (g/L) | 38.45 ± 7.06 | 46.77 ± 3.57 | < 0.001 |
| Globulin ^a^ (g/L) | 33.55 ± 10.39 | 28.07 ± 11.85 | < 0.001 |
| Glucose ^a^ (mmol/L) | 5.58 ± 2.23 | 4.93 ± 0.65 | < 0.001 |
| Alanine transaminase ^b^ (IU/L) | 17.00 (11.00 - 30.00) | 19.00 (14.00 - 29.00) | < 0.001 |
| Aspartate transaminase ^b^ (IU/L) | 23.00 (17.00 - 32.00) | 21.00 (18.00 - 25.00) | < 0.001 |
| Gamma-glutamyl transpeptidase ^b^ (IU/L) | 32.00 (18.00 - 58.75) | 14.00 (10.00 - 24.00) | < 0.001 |
| Alkaline phosphatase ^b^ (IU/L) | 84.00 (68.00 - 108.00) | 68.00 (56.00 - 82.00) | < 0.001 |
| Total bilirubin ^b^ (μmol/L) | 9.40 (6.60 - 13.50) | 12.60 (9.03 - 16.20) | < 0.001 |
| Direct bilirubin ^b^ (μmol/L) | 3.70 (2.60 - 5.70) | 3.80 (2.60 - 5.00) | 0.009 |
| Indirect bilirubin ^b^ (μmol/L) | 5.20 (3.60 - 7.70) | 8.70 (6.10 - 11.20) | < 0.001 |
| Urea nitrogen ^b^ (mmol/L) | 4.30 (3.33 - 5.50) | 4.90 (4.00 - 6.20) | < 0.001 |
| Creatinine ^b^ (μmol/L) | 61.00 (50.00 - 73.00) | 68.00 (55.00 - 80.00) | < 0.001 |
| Cystatin-C ^b^ (mg/L) | 0.91 (0.78 - 1.06) | 0.81 (0.73 - 0.94) | < 0.001 |

^a^ The results were displayed as mean ± standard deviations, and tested by t-test; ^b^ The results were displayed as median (interquartile range) values and tested by Mann-Whitney U test.

**Table S2. Laboratory indexes of the tuberculosis patients.**

| **Laboratory indexes** | **Han (n = 1532)** | **Tibetan (n = 211)** | ***P*** |
| --- | --- | --- | --- |
| Hematocrit ^a^ (%) | 0.38 ± 0.06 | 0.38 ± 0.08 | 0.747 |
| Hemoglobin ^a^ (g/L) | 124.16 ± 22.62 | 126.06 ± 28.24 | 0.441 |
| Erythrocyte ^a^ (×10^12^/L) | 4.34 ± 0.73 | 4.48 ± 0.98 | 0.102 |
| Platelet ^a^ (×10^9^/L) | 255.85 ± 128.99 | 269.96 ± 131.50 | 0.222 |
| Leukocyte ^a^ (×10^9^/L) | 7.44 ± 3.41 | 6.64 ± 3.04 | 0.008 |
| Monocyte ^a^ (×10^9^/L) | 0.50 ± 0.27 | 0.46 ± 0.27 | 0.059 |
| Albumin ^a^ (g/L) | 38.88 ± 7.01 | 36.33 ± 7.04 | < 0.001 |
| Globulin ^a^ (g/L) | 32.82 ± 8.90 | 38.37 ± 16.56 | < 0.001 |
| Glucose ^a^ (mmol/L) | 5.18 ± 1.46 | 5.42 ± 2.42 | 0.272 |
| Alanine transaminase ^b^ (IU/L) | 17.00 (11.00 - 30.00) | 17.00 (10.50 - 33.00) | 0.888 |
| Aspartate transaminase ^b^ (IU/L) | 23.00 (17.00 - 32.00) | 22.00 (17.00 - 35.00) | 0.867 |
| Gamma-glutamyl transpeptidase ^b^ (IU/L) | 29.00 (17.00 - 56.00) | 45.00 (26.50 - 87.50) | < 0.001 |
| Alkaline phosphatase ^b^ (IU/L) | 82.00 (66.00 - 104.00) | 100.00 (79.50 - 140.50) | < 0.001 |
| Total bilirubin ^b^ (μmol/L) | 9.50 (6.90 - 13.50) | 8.30 (5.40 - 13.95) | 0.019 |
| Direct bilirubin ^b^ (μmol/L) | 3.70 (2.60 - 5.60) | 3.70 (2.40 - 5.90) | 0.867 |
| Indirect bilirubin ^b^ (μmol/L) | 5.40 (3.70 - 7.80) | 4.60 (2.60 - 6.55) | < 0.001 |
| Urea nitrogen ^b^ (mmol/L) | 4.31 (3.40 - 5.50) | 4.00 (3.00 - 5.65) | 0.059 |
| Creatinine ^b^ (μmol/L) | 62.00 (51.00 - 74.00) | 53.00 (42.00 - 67.00) | < 0.001 |
| Cystatin-C ^b^ (mg/L) | 0.91 (0.78 - 1.06) | 0.85 (0.75 - 1.02) | 0.045 |
| C-reactive protein ^b^ (mg/L) | 14.75 (3.56 - 55.75) | 17.15 (5.53 - 62.18) | 0.181 |
| Erythrocyte sedimentation rate ^b^ (mm/h) | 44.00 (21.00 - 68.75) | 40.00 (15.00 - 65.00) | 0.110 |

^a^ The results were displayed as mean ± standard deviations, and tested by t-test; ^b^ The results were displayed as median (interquartile range) values and tested by Mann-Whitney U test.

**Table S3. SNPs with *P* < 1 × 10^−5^ in the Chinese Han population.**

| **Chr** | **SNP** | **Position** | **Effect allele** | **F_TB** | **F_HC** | **Unadjusted** | | | | **Adjusted ^#^** | | | | |
| --- | --- | --- | --- | --- | --- | --- | --- | --- | --- | --- | --- | --- | --- | --- |
|  |  |  |  |  |  | **OR** | **95% CI** | **SE** | ***P*** | **OR** | **95% CI** | **SE** | ***P*** | ***P*__Conditioned_** |
| **2** | **rs7607919** | **2076520** | **A** | **0.069** | **0.032** | **2.263** | **1.772-2.891** | **0.125** | **2.10E-11** | **1.980** | **1.528-2.565** | **0.132** | **2.36E-07** | **NA** |
| 2 | rs76840858 | 2082664 | C | 0.068 | 0.031 | 2.257 | 1.767-2.883 | 0.125 | 2.54E-11 | 1.969 | 1.519-2.551 | 0.132 | 3.01E-07 | NA |
| 2 | rs76688230 | 2090469 | T | 0.068 | 0.031 | 2.257 | 1.766-2.883 | 0.125 | 2.55E-11 | 1.967 | 1.518-2.549 | 0.132 | 3.09E-07 | NA |
| 2 | rs74973125 | 2086567 | A | 0.067 | 0.031 | 2.232 | 1.746-2.852 | 0.125 | 5.21E-11 | 1.958 | 1.510-2.539 | 0.133 | 3.90E-07 | NA |
| 2 | rs76123245 | 2086720 | T | 0.067 | 0.031 | 2.232 | 1.746-2.852 | 0.125 | 5.21E-11 | 1.958 | 1.510-2.539 | 0.133 | 3.90E-07 | NA |
| 2 | rs142466632 | 2067549 | A | 0.067 | 0.031 | 2.220 | 1.737-2.837 | 0.125 | 7.26E-11 | 1.957 | 1.509-2.537 | 0.133 | 4.03E-07 | NA |
| 2 | rs77784026 | 2061411 | A | 0.069 | 0.033 | 2.130 | 1.677-2.705 | 0.122 | 2.48E-10 | 1.892 | 1.469-2.438 | 0.129 | 8.20E-07 | NA |
| 2 | rs73911366 | 2115283 | T | 0.067 | 0.031 | 2.244 | 1.752-2.875 | 0.126 | 5.77E-11 | 1.916 | 1.474-2.491 | 0.134 | 1.19E-06 | NA |
| 2 | rs75609205 | 2159804 | A | 0.071 | 0.032 | 2.327 | 1.826-2.965 | 0.124 | 2.51E-12 | 1.963 | 1.516-2.543 | 0.132 | 3.19E-07 | 0.740 |
| 2 | rs73913052 | 2058880 | C | 0.070 | 0.034 | 2.117 | 1.670-2.683 | 0.121 | 2.53E-10 | 1.859 | 1.445-2.392 | 0.129 | 1.44E-06 | 0.961 |
| 2 | rs78980377 | 2115581 | T | 0.065 | 0.030 | 2.199 | 1.713-2.822 | 0.127 | 2.52E-10 | 1.890 | 1.450-2.464 | 0.135 | 2.55E-06 | 0.275 |
| **2** | **rs191940547** | **22947152** | **G** | **0.019** | **0.005** | **3.801** | **2.181-6.626** | **0.284** | **4.26E-07** | **4.054** | **2.278-7.214** | **0.294** | **1.94E-06** | **NA** |
| **5** | **rs6876373** | **145376268** | **G** | **0.266** | **0.322** | **0.761** | **0.682-0.850** | **0.056** | **1.14E-06** | **0.761** | **0.678-0.854** | **0.059** | **3.58E-06** | **NA** |
| 5 | rs17426347 | 145370681 | A | 0.266 | 0.323 | 0.760 | 0.681-0.849 | 0.056 | 1.05E-06 | 0.762 | 0.679-0.855 | 0.059 | 3.92E-06 | NA |
| 5 | rs17492228 | 145377479 | C | 0.266 | 0.322 | 0.765 | 0.685-0.854 | 0.056 | 1.68E-06 | 0.764 | 0.681-0.858 | 0.059 | 5.01E-06 | NA |
| 5 | rs17426578 | 145377578 | C | 0.266 | 0.322 | 0.765 | 0.685-0.854 | 0.056 | 1.68E-06 | 0.764 | 0.681-0.858 | 0.059 | 5.01E-06 | NA |
| 5 | rs1558147 | 145377965 | A | 0.266 | 0.322 | 0.765 | 0.685-0.854 | 0.056 | 1.68E-06 | 0.764 | 0.681-0.858 | 0.059 | 5.01E-06 | NA |
| 5 | rs6898375 | 145376310 | A | 0.266 | 0.321 | 0.765 | 0.685-0.854 | 0.056 | 1.80E-06 | 0.765 | 0.681-0.858 | 0.059 | 5.15E-06 | NA |
| 5 | rs4912703 | 145372622 | C | 0.266 | 0.322 | 0.765 | 0.685-0.854 | 0.056 | 1.75E-06 | 0.765 | 0.681-0.858 | 0.059 | 5.24E-06 | NA |
| 5 | rs4913060 | 145377136 | T | 0.266 | 0.322 | 0.765 | 0.686-0.854 | 0.056 | 1.83E-06 | 0.765 | 0.682-0.859 | 0.059 | 5.35E-06 | NA |
| 5 | rs4141240 | 145371192 | G | 0.266 | 0.322 | 0.765 | 0.685-0.854 | 0.056 | 1.75E-06 | 0.765 | 0.682-0.859 | 0.059 | 5.41E-06 | NA |
| 5 | rs977204 | 145371320 | A | 0.266 | 0.322 | 0.765 | 0.685-0.854 | 0.056 | 1.75E-06 | 0.765 | 0.682-0.859 | 0.059 | 5.41E-06 | NA |
| 5 | rs17426499 | 145377423 | T | 0.264 | 0.320 | 0.765 | 0.685-0.854 | 0.056 | 1.80E-06 | 0.765 | 0.681-0.859 | 0.059 | 5.60E-06 | NA |
| 5 | rs6893852 | 145372207 | G | 0.265 | 0.320 | 0.764 | 0.684-0.853 | 0.056 | 1.77E-06 | 0.765 | 0.681-0.859 | 0.059 | 5.71E-06 | NA |
| 5 | rs6876582 | 145376462 | G | 0.264 | 0.320 | 0.765 | 0.685-0.854 | 0.056 | 1.92E-06 | 0.765 | 0.681-0.859 | 0.059 | 5.75E-06 | NA |
| 5 | rs2895646 | 145381737 | G | 0.266 | 0.321 | 0.766 | 0.687-0.855 | 0.056 | 1.85E-06 | 0.766 | 0.683-0.860 | 0.059 | 5.88E-06 | NA |
| 5 | rs9325000 | 145380270 | C | 0.265 | 0.320 | 0.765 | 0.686-0.854 | 0.056 | 1.82E-06 | 0.766 | 0.682-0.859 | 0.059 | 5.91E-06 | NA |
| 5 | rs2400207 | 145380097 | A | 0.266 | 0.321 | 0.766 | 0.687-0.855 | 0.056 | 1.96E-06 | 0.766 | 0.683-0.860 | 0.059 | 5.97E-06 | NA |
| 5 | rs977205 | 145371780 | C | 0.267 | 0.323 | 0.765 | 0.685-0.854 | 0.056 | 1.90E-06 | 0.766 | 0.682-0.860 | 0.059 | 6.48E-06 | NA |
| 5 | rs12652801 | 145376757 | C | 0.264 | 0.320 | 0.765 | 0.684-0.854 | 0.057 | 1.99E-06 | 0.766 | 0.682-0.860 | 0.059 | 6.58E-06 | NA |
| 5 | rs2159108 | 145380618 | C | 0.266 | 0.321 | 0.768 | 0.688-0.857 | 0.056 | 2.27E-06 | 0.768 | 0.684-0.862 | 0.059 | 7.02E-06 | NA |
| 5 | rs1158395 | 145381669 | T | 0.265 | 0.319 | 0.768 | 0.688-0.858 | 0.056 | 2.63E-06 | 0.769 | 0.685-0.863 | 0.059 | 8.33E-06 | NA |
| 5 | rs12522478 | 145385898 | G | 0.267 | 0.322 | 0.768 | 0.689-0.857 | 0.056 | 2.32E-06 | 0.771 | 0.688-0.865 | 0.058 | 8.88E-06 | NA |
| **5** | **rs78023096** | **73215056** | **A** | **0.045** | **0.069** | **0.636** | **0.511-0.792** | **0.112** | **4.67E-05** | **0.586** | **0.463-0.742** | **0.120** | **9.24E-06** | **NA** |
| 5 | rs2312936 | 73213163 | T | 0.045 | 0.069 | 0.635 | 0.510-0.791 | 0.112 | 4.29E-05 | 0.586 | 0.463-0.743 | 0.120 | 9.27E-06 | 0.911 |
| **6** | **rs111875628** | **32583813** | **A** | **0.293** | **0.243** | **1.294** | **1.157-1.449** | **0.057** | **6.86E-06** | **1.337** | **1.185-1.508** | **0.061** | **2.24E-06** | **NA** |
| **7** | **rs8180820** | **51964546** | **C** | **0.509** | **0.442** | **1.305** | **1.179-1.445** | **0.052** | **2.77E-07** | **1.304** | **1.172-1.452** | **0.055** | **1.18E-06** | **NA** |
| 7 | rs74379784 | 51966126 | G | 0.507 | 0.444 | 1.288 | 1.164-1.425 | 0.052 | 8.96E-07 | 1.287 | 1.157-1.431 | 0.054 | 3.37E-06 | NA |
| 7 | rs2329848 | 51966872 | A | 0.505 | 0.443 | 1.286 | 1.163-1.423 | 0.051 | 9.03E-07 | 1.284 | 1.155-1.427 | 0.054 | 3.65E-06 | NA |
| 7 | rs997556 | 51964745 | T | 0.504 | 0.439 | 1.299 | 1.173-1.438 | 0.052 | 4.82E-07 | 1.301 | 1.168-1.449 | 0.055 | 1.73E-06 | 0.056 |
| 7 | rs4947648 | 51968017 | G | 0.503 | 0.441 | 1.281 | 1.159-1.416 | 0.051 | 1.25E-06 | 1.289 | 1.160-1.432 | 0.054 | 2.34E-06 | 0.897 |
| 7 | rs76093377 | 51969209 | C | 0.505 | 0.443 | 1.286 | 1.163-1.421 | 0.051 | 7.98E-07 | 1.284 | 1.156-1.426 | 0.054 | 3.06E-06 | 0.818 |
| 7 | rs56805182 | 51972775 | G | 0.505 | 0.443 | 1.284 | 1.162-1.419 | 0.051 | 9.07E-07 | 1.283 | 1.155-1.425 | 0.054 | 3.29E-06 | 0.615 |
| 7 | rs12669031 | 51979948 | C | 0.506 | 0.443 | 1.284 | 1.161-1.419 | 0.051 | 9.74E-07 | 1.283 | 1.155-1.425 | 0.054 | 3.50E-06 | 0.843 |
| 7 | rs17151293 | 51973256 | C | 0.504 | 0.442 | 1.283 | 1.161-1.417 | 0.051 | 9.50E-07 | 1.281 | 1.154-1.423 | 0.053 | 3.58E-06 | 0.965 |
| 7 | rs1993956 | 51970823 | T | 0.502 | 0.443 | 1.271 | 1.151-1.404 | 0.051 | 2.33E-06 | 1.279 | 1.152-1.42 | 0.053 | 4.00E-06 | 0.630 |
| 7 | rs2329851 | 51976239 | G | 0.505 | 0.444 | 1.282 | 1.160-1.416 | 0.051 | 1.12E-06 | 1.280 | 1.153-1.422 | 0.054 | 4.05E-06 | 0.844 |
| 7 | rs4947650 | 51970769 | A | 0.501 | 0.441 | 1.273 | 1.152-1.406 | 0.051 | 2.26E-06 | 1.278 | 1.151-1.420 | 0.054 | 4.64E-06 | 0.916 |
| **7** | **rs886682** | **48872717** | **C** | **0.422** | **0.467** | **0.835** | **0.754-0.923** | **0.051** | **4.37E-04** | **0.786** | **0.707-0.873** | **0.054** | **6.56E-06** | **NA** |
| 7 | rs6421316 | 48871374 | G | 0.421 | 0.466 | 0.834 | 0.753-0.922 | 0.052 | 4.05E-04 | 0.788 | 0.709-0.875 | 0.054 | 8.13E-06 | 0.684 |
| **7** | **rs62454380** | **48885455** | **T** | **0.258** | **0.211** | **1.299** | **1.152-1.466** | **0.061** | **1.99E-05** | **1.351** | **1.189-1.535** | **0.065** | **3.76E-06** | **NA** |
| 7 | rs11532820 | 48887661 | A | 0.265 | 0.218 | 1.294 | 1.150-1.456 | 0.060 | 1.78E-05 | 1.331 | 1.174-1.508 | 0.064 | 7.61E-06 | NA |
| 7 | rs62454386 | 48886783 | A | 0.261 | 0.214 | 1.303 | 1.156-1.467 | 0.061 | 1.34E-05 | 1.338 | 1.179-1.519 | 0.065 | 6.60E-06 | 0.879 |
| **8** | **rs146050799** | **95307788** | **T** | **0.070** | **0.046** | **1.573** | **1.260-1.964** | **0.113** | **5.65E-05** | **1.760** | **1.388-2.233** | **0.121** | **3.12E-06** | **NA** |
| 8 | rs74395058 | 95302283 | A | 0.072 | 0.049 | 1.509 | 1.217-1.871 | 0.110 | 1.65E-04 | 1.682 | 1.337-2.117 | 0.117 | 9.31E-06 | 0.911 |
| **10** | **rs117041426** | **91108040** | **A** | **0.059** | **0.090** | **0.635** | **0.522-0.772** | **0.100** | **4.47E-06** | **0.624** | **0.507-0.769** | **0.106** | **9.24E-06** | **NA** |
| 10 | rs118043504 | 91108041 | T | 0.059 | 0.090 | 0.635 | 0.522-0.772 | 0.100 | 4.47E-06 | 0.624 | 0.507-0.769 | 0.106 | 9.24E-06 | NA |
| **15** | **rs62045225** | **56271008** | **C** | **0.146** | **0.110** | **1.383** | **1.190-1.607** | **0.077** | **2.26E-05** | **1.452** | **1.238-1.702** | **0.081** | **4.48E-06** | **NA** |
| 15 | rs117927944 | 56271524 | G | 0.143 | 0.108 | 1.374 | 1.181-1.599 | 0.077 | 3.76E-05 | 1.443 | 1.229-1.694 | 0.082 | 7.43E-06 | 0.857 |
| **18** | **rs62094793** | **21402075** | **G** | **0.040** | **0.021** | **1.962** | **1.438-2.676** | **0.159** | **1.52E-05** | **2.195** | **1.580-3.049** | **0.168** | **2.74E-06** | **NA** |
| 18 | rs12458273 | 21371052 | T | 0.039 | 0.021 | 1.883 | 1.384-2.561 | 0.157 | 4.35E-05 | 2.127 | 1.537-2.944 | 0.166 | 5.35E-06 | 0.945 |
| **18** | **rs12717067** | **65209837** | **T** | **0.425** | **0.371** | **1.254** | **1.131-1.390** | **0.053** | **1.71E-05** | **1.297** | **1.161-1.449** | **0.057** | **4.16E-06** | **NA** |
| 18 | rs8095562 | 65210224 | C | 0.425 | 0.371 | 1.251 | 1.128-1.387 | 0.053 | 2.03E-05 | 1.295 | 1.159-1.447 | 0.056 | 4.66E-06 | NA |
| 18 | rs9953551 | 65210775 | G | 0.425 | 0.372 | 1.248 | 1.126-1.384 | 0.053 | 2.41E-05 | 1.292 | 1.157-1.444 | 0.056 | 5.52E-06 | NA |
| 18 | rs9957826 | 65208280 | C | 0.395 | 0.340 | 1.269 | 1.143-1.409 | 0.053 | 7.69E-06 | 1.296 | 1.158-1.452 | 0.058 | 7.02E-06 | 0.878 |
| **20** | **rs200331599** | **35634297** | **T** | **0.003** | **0.020** | **0.131** | **0.063-0.274** | **0.377** | **2.14E-10** | **0.103** | **0.047-0.226** | **0.403** | **1.60E-08** | **NA** |

The chromosomal positions are based on NCBI Build 37. Chr: chromosome; F_TB: frequency of tuberculosis; F_HC: frequency of healthy controls; OR: odds ratio; CI: confidence interval; SE: standard error. ^#^ The result was tested by the additive model and adjusted by eight significant principal components, age, and sex. *P*__Conditioned_ was the *P* value for conditional analysis on the lead SNP in bold.

**Table S4. SNPs with *P* < 1 × 10^−5^ in the Chinese Tibetan population.**

| **Chr** | **SNP** | **Position** | **Effect allele** | **F_TB** | **F_HC** | **Unadjusted** | | | | **Adjusted ^#^** | | | | |
| --- | --- | --- | --- | --- | --- | --- | --- | --- | --- | --- | --- | --- | --- | --- |
|  |  |  |  |  |  | **OR** | **95% CI** | **SE** | ***P*** | **OR** | **95% CI** | **SE** | ***P*** | ***P*__Conditioned_** |
| **2** | **rs12465906** | **165032962** | **T** | **0.080** | **0.034** | **2.502** | **1.338-4.678** | **0.319** | **3.09E-03** | **8.007** | **3.419-18.750** | **0.434** | **1.65E-06** | **NA** |
| 2 | rs734529 | 165027707 | T | 0.075 | 0.032 | 2.474 | 1.297-4.721 | 0.330 | 4.65E-03 | 8.640 | 3.490-21.390 | 0.463 | 3.12E-06 | 0.983 |
| 2 | rs2034256 | 165026230 | T | 0.073 | 0.033 | 2.331 | 1.240-4.382 | 0.322 | 7.03E-03 | 8.360 | 3.417-20.450 | 0.456 | 3.28E-06 | 0.984 |
| 2 | rs6738278 | 165026936 | T | 0.073 | 0.033 | 2.331 | 1.240-4.382 | 0.322 | 7.03E-03 | 8.360 | 3.417-20.450 | 0.456 | 3.28E-06 | 0.984 |
| 2 | rs6432781 | 165028592 | G | 0.073 | 0.033 | 2.331 | 1.240-4.382 | 0.322 | 7.03E-03 | 8.360 | 3.417-20.450 | 0.456 | 3.28E-06 | 0.984 |
| 2 | rs7606186 | 165028618 | A | 0.073 | 0.033 | 2.331 | 1.240-4.382 | 0.322 | 7.03E-03 | 8.360 | 3.417-20.450 | 0.456 | 3.28E-06 | 0.984 |
| 2 | rs6432782 | 165029043 | C | 0.073 | 0.033 | 2.331 | 1.240-4.382 | 0.322 | 7.03E-03 | 8.360 | 3.417-20.450 | 0.456 | 3.28E-06 | 0.984 |
| 2 | rs72875856 | 165029474 | T | 0.073 | 0.033 | 2.331 | 1.240-4.382 | 0.322 | 7.03E-03 | 8.360 | 3.417-20.450 | 0.456 | 3.28E-06 | 0.984 |
| 2 | rs72875857 | 165029546 | A | 0.073 | 0.033 | 2.331 | 1.240-4.382 | 0.322 | 7.03E-03 | 8.360 | 3.417-20.450 | 0.456 | 3.28E-06 | 0.984 |
| 2 | rs1838830 | 165030119 | T | 0.073 | 0.033 | 2.331 | 1.240-4.382 | 0.322 | 7.03E-03 | 8.360 | 3.417-20.450 | 0.456 | 3.28E-06 | 0.984 |
| 2 | rs1838831 | 165030132 | C | 0.073 | 0.033 | 2.331 | 1.240-4.382 | 0.322 | 7.03E-03 | 8.360 | 3.417-20.450 | 0.456 | 3.28E-06 | 0.984 |
| 2 | rs12468763 | 165030467 | A | 0.073 | 0.033 | 2.331 | 1.240-4.382 | 0.322 | 7.03E-03 | 8.360 | 3.417-20.450 | 0.456 | 3.28E-06 | 0.984 |
| 2 | rs2084188 | 165031054 | G | 0.073 | 0.033 | 2.331 | 1.240-4.382 | 0.322 | 7.03E-03 | 8.360 | 3.417-20.450 | 0.456 | 3.28E-06 | 0.984 |
| 2 | rs2084189 | 165031141 | T | 0.073 | 0.033 | 2.331 | 1.240-4.382 | 0.322 | 7.03E-03 | 8.360 | 3.417-20.450 | 0.456 | 3.28E-06 | 0.984 |
| 2 | rs7587276 | 165032389 | A | 0.073 | 0.033 | 2.331 | 1.240-4.382 | 0.322 | 7.03E-03 | 8.360 | 3.417-20.450 | 0.456 | 3.28E-06 | 0.984 |
| 2 | rs7587099 | 165032419 | T | 0.073 | 0.033 | 2.331 | 1.240-4.382 | 0.322 | 7.03E-03 | 8.360 | 3.417-20.450 | 0.456 | 3.28E-06 | 0.984 |
| 2 | rs7587194 | 165032472 | A | 0.073 | 0.033 | 2.331 | 1.240-4.382 | 0.322 | 7.03E-03 | 8.360 | 3.417-20.450 | 0.456 | 3.28E-06 | 0.984 |
| 2 | rs12470215 | 165032924 | G | 0.073 | 0.033 | 2.331 | 1.240-4.382 | 0.322 | 7.03E-03 | 8.360 | 3.417-20.450 | 0.456 | 3.28E-06 | 0.984 |
| 2 | rs72875870 | 165033006 | A | 0.073 | 0.033 | 2.331 | 1.240-4.382 | 0.322 | 7.03E-03 | 8.360 | 3.417-20.450 | 0.456 | 3.28E-06 | 0.984 |
| 2 | rs34295647 | 165035044 | C | 0.073 | 0.033 | 2.331 | 1.240-4.382 | 0.322 | 7.03E-03 | 8.360 | 3.417-20.450 | 0.456 | 3.28E-06 | 0.984 |
| 2 | rs12468233 | 165035145 | A | 0.073 | 0.033 | 2.331 | 1.240-4.382 | 0.322 | 7.03E-03 | 8.360 | 3.417-20.450 | 0.456 | 3.28E-06 | 0.984 |
| 2 | rs12469350 | 165035861 | A | 0.073 | 0.033 | 2.331 | 1.240-4.382 | 0.322 | 7.03E-03 | 8.360 | 3.417-20.450 | 0.456 | 3.28E-06 | 0.984 |
| 2 | rs1446466 | 165035887 | T | 0.073 | 0.033 | 2.331 | 1.240-4.382 | 0.322 | 7.03E-03 | 8.360 | 3.417-20.450 | 0.456 | 3.28E-06 | 0.984 |
| 2 | rs17352150 | 165036412 | A | 0.073 | 0.033 | 2.331 | 1.240-4.382 | 0.322 | 7.03E-03 | 8.360 | 3.417-20.450 | 0.456 | 3.28E-06 | 0.984 |
| 2 | rs72875889 | 165036531 | A | 0.073 | 0.033 | 2.331 | 1.240-4.382 | 0.322 | 7.03E-03 | 8.360 | 3.417-20.450 | 0.456 | 3.28E-06 | 0.984 |
| 2 | rs6757401 | 165036762 | A | 0.073 | 0.033 | 2.331 | 1.240-4.382 | 0.322 | 7.03E-03 | 8.360 | 3.417-20.450 | 0.456 | 3.28E-06 | 0.984 |
| 2 | rs6715136 | 165036779 | C | 0.073 | 0.033 | 2.331 | 1.240-4.382 | 0.322 | 7.03E-03 | 8.360 | 3.417-20.450 | 0.456 | 3.28E-06 | 0.984 |
| 2 | rs12470648 | 165037295 | T | 0.073 | 0.033 | 2.331 | 1.240-4.382 | 0.322 | 7.03E-03 | 8.360 | 3.417-20.450 | 0.456 | 3.28E-06 | 0.984 |
| 2 | rs12470726 | 165037553 | T | 0.073 | 0.033 | 2.331 | 1.240-4.382 | 0.322 | 7.03E-03 | 8.360 | 3.417-20.450 | 0.456 | 3.28E-06 | 0.984 |
| 2 | rs72875896 | 165039607 | G | 0.073 | 0.033 | 2.331 | 1.240-4.382 | 0.322 | 7.03E-03 | 8.360 | 3.417-20.450 | 0.456 | 3.28E-06 | 0.984 |
| 2 | rs1439218 | 165040140 | A | 0.073 | 0.033 | 2.331 | 1.240-4.382 | 0.322 | 7.03E-03 | 8.360 | 3.417-20.450 | 0.456 | 3.28E-06 | 0.984 |
| 2 | rs17436698 | 165041174 | C | 0.073 | 0.033 | 2.331 | 1.240-4.382 | 0.322 | 7.03E-03 | 8.360 | 3.417-20.450 | 0.456 | 3.28E-06 | 0.984 |
| 2 | rs1823113 | 165031246 | A | 0.073 | 0.033 | 2.310 | 1.228-4.343 | 0.322 | 7.71E-03 | 8.339 | 3.407-20.410 | 0.457 | 3.41E-06 | 0.986 |
| 2 | rs12468143 | 165034745 | A | 0.073 | 0.033 | 2.310 | 1.228-4.343 | 0.322 | 7.71E-03 | 8.276 | 3.383-20.240 | 0.456 | 3.64E-06 | NA |
| 2 | rs2084191 | 165031318 | G | 0.067 | 0.029 | 2.448 | 1.250-4.793 | 0.343 | 7.17E-03 | 8.994 | 3.497-23.130 | 0.482 | 5.17E-06 | 0.965 |
| 2 | rs12468111 | 165034645 | A | 0.071 | 0.033 | 2.262 | 1.199-4.266 | 0.324 | 9.85E-03 | 7.969 | 3.227-19.680 | 0.461 | 6.79E-06 | 0.986 |
| 2 | rs17436622 | 165035609 | G | 0.071 | 0.033 | 2.262 | 1.199-4.266 | 0.324 | 9.85E-03 | 7.969 | 3.227-19.680 | 0.461 | 6.79E-06 | 0.986 |
| 2 | rs72875887 | 165036513 | T | 0.071 | 0.033 | 2.262 | 1.199-4.266 | 0.324 | 9.85E-03 | 7.969 | 3.227-19.680 | 0.461 | 6.79E-06 | 0.986 |
| 2 | rs115734710 | 165030671 | A | 0.071 | 0.033 | 2.251 | 1.193-4.247 | 0.324 | 1.03E-02 | 7.913 | 3.207-19.530 | 0.461 | 7.18E-06 | NA |
| **7** | **rs7802755** | **67564211** | **A** | **0.527** | **0.400** | **1.672** | **1.276-2.192** | **0.138** | **1.86E-04** | **2.361** | **1.637-3.407** | **0.187** | **4.33E-06** | **NA** |
| 7 | rs12698667 | 67565028 | C | 0.527 | 0.402 | 1.657 | 1.264-2.171 | 0.138 | 2.42E-04 | 2.351 | 1.628-3.396 | 0.188 | 5.22E-06 | NA |
| 7 | rs63717762 | 67565704 | T | 0.527 | 0.402 | 1.657 | 1.264-2.171 | 0.138 | 2.42E-04 | 2.351 | 1.628-3.396 | 0.188 | 5.22E-06 | NA |
| 7 | rs4718709 | 67567429 | G | 0.528 | 0.404 | 1.657 | 1.268-2.164 | 0.137 | 2.08E-04 | 2.323 | 1.614-3.344 | 0.186 | 5.77E-06 | NA |
| 7 | rs12698665 | 67561951 | T | 0.524 | 0.400 | 1.656 | 1.264-2.170 | 0.138 | 2.46E-04 | 2.333 | 1.619-3.361 | 0.186 | 5.44E-06 | 0.973 |
| 7 | rs6958198 | 67561265 | C | 0.524 | 0.400 | 1.657 | 1.264-2.172 | 0.138 | 2.47E-04 | 2.331 | 1.618-3.358 | 0.186 | 5.50E-06 | 0.974 |
| 7 | rs6949199 | 67560687 | G | 0.525 | 0.400 | 1.658 | 1.264-2.174 | 0.138 | 2.48E-04 | 2.327 | 1.616-3.351 | 0.186 | 5.67E-06 | 0.975 |
| 7 | rs4718708 | 67560787 | T | 0.525 | 0.400 | 1.658 | 1.264-2.174 | 0.138 | 2.48E-04 | 2.327 | 1.616-3.351 | 0.186 | 5.67E-06 | 0.975 |
| 7 | rs6957409 | 67566119 | T | 0.535 | 0.409 | 1.662 | 1.265-2.184 | 0.139 | 2.53E-04 | 2.344 | 1.619-3.391 | 0.189 | 6.27E-06 | 0.586 |
| 7 | rs13246932 | 67562946 | T | 0.507 | 0.383 | 1.656 | 1.262-2.173 | 0.139 | 2.66E-04 | 2.272 | 1.581-3.263 | 0.185 | 9.03E-06 | 0.508 |
| 7 | rs12698666 | 67563175 | A | 0.507 | 0.383 | 1.656 | 1.262-2.173 | 0.139 | 2.66E-04 | 2.272 | 1.581-3.263 | 0.185 | 9.03E-06 | 0.508 |
| **12** | **rs73067158** | **18368458** | **T** | **0.089** | **0.188** | **0.424** | **0.281-0.640** | **0.210** | **3.05E-05** | **0.303** | **0.179-0.513** | **0.269** | **8.95E-06** | **NA** |

The chromosomal positions are based on NCBI Build 37. Chr: chromosome; F_TB: frequency of tuberculosis; F_HC: frequency of healthy controls; OR: odds ratio; CI: confidence interval; SE: standard error. ^#^ The result was tested by the additive model and adjusted by five significant principal components, age, and sex. *P*__Conditioned_ was the *P* value for conditional analysis on the lead SNP in bold.

**Table S5. Performance of previously reported SNPs in the present population.**

| **Chr** | **SNP** | **Position** | **Previous reports** | | | | | **Chinese Han population** | | | | | **Chinese Tibetan population** | | | | |
| --- | --- | --- | --- | --- | --- | --- | --- | --- | --- | --- | --- | --- | --- | --- | --- | --- | --- |
|  |  |  | **Effect allele** | **F_HC** | **OR** | **95% CI** | ***P*** | **Minor allele** | **F_HC** | **OR** | **95% CI** | ***P*** | **Minor allele** | **F_HC** | **OR** | **95% CI** | ***P*** |
| 1 | rs4240897 ^[12]^ | 12042755 | A | 0.507 | 0.79 | 0.75-0.83 | 1.41E-11 | G | 0.463 | 1.07 | 0.96-1.19 | 0.229 | G | 0.428 | 1.29 | 0.90-1.85 | 0.166 |
| 4 | rs2269497 ^[12]^ | 3429856 | G | 0.056 | 1.51 | 1.35-1.68 | 3.37E-08 | G | 0.055 | 0.99 | 0.78-1.24 | 0.899 | G | 0.075 | 1.55 | 0.87-2.76 | 0.136 |
| 6 | rs41553512 ^[12]^ | 32486402 | A | 0.016 | 2.14 | 1.78-2.57 | 7.93E-11 | T | 0.023 | 1.23 | 0.88-1.73 | 0.224 | T | 0.004 | 3.38 | 0.42-27.07 | 0.252 |
| 6 | rs557011 ^[11]^ | 32587013 | T | 0.402 | 1.25 | 1.17-1.33 | 5.80E-12 | C | 0.478 | 0.84 | 0.75-0.94 | **0.002** | T | 0.404 | 1.62 | 1.13-2.32 | **0.009** |
| 6 | rs9271378 ^[11]^ | 32587300 | G | 0.325 | 0.78 | 0.73-0.84 | 2.50E-12 | G | 0.240 | 1.00 | 0.87-1.15 | 0.951 | G | 0.386 | 0.73 | 0.50-1.06 | 0.096 |
| 6 | rs9272785 ^[11]^ | 32610401 | A | 0.191 | 1.22 | 1.13-1.32 | 3.50E-07 | A | 0.278 | 1.10 | 0.93-1.29 | 0.260 | A | 0.281 | 1.54 | 1.06-2.24 | **0.024** |
| 8 | rs10956514 ^[10]^ | 131252758 | G | 0.358 | 0.85 | 0.81-0.89 | 1.00E-10 | A | 0.368 | 1.06 | 0.95-1.19 | 0.275 | A | 0.467 | 0.98 | 0.70-1.38 | 0.918 |
| 8 | rs4733781 ^[10]^ | 131296767 | C | 0.314 | 0.84 | 0.80-0.88 | 2.60E-11 | A | 0.378 | 1.07 | 0.96-1.19 | 0.227 | A | 0.480 | 1.01 | 0.72-1.41 | 0.970 |
| 11 | rs2057178 ^[9]^ | 32364187 | A | 0.110-0.320 | 0.82 | 0.77-0.86 | 2.57E-11 | A | 0.055 | 0.76 | 0.59-0.97 | **0.029** | A | 0.059 | 0.99 | 0.44-2.21 | 0.978 |
| 18 | rs4331426 ^[8]^ | 20190795 | G | 0.429-0.525 | 1.19 | 1.13-1.27 | 6.80E-09 | G | 0.028 | 1.24 | 0.90-1.69 | 0.184 | G | 0.024 | 0.67 | 0.19-2.35 | 0.534 |

The chromosomal positions are based on NCBI Build 37. Chr: chromosome; F_HC: frequency of healthy controls; OR: odds ratio; CI: confidence interval.

**Table S6. The eQTL and sQTL effects of suggestive association loci.**

| **Chr** | **SNP** | **Position** | **Effect allele** | **Single-tissue eQTL hits** | **Single-tissue sQTL hits** |
| --- | --- | --- | --- | --- | --- |
| 2 | rs6544900 | 46752101 | A | 34 | 0 |
| 5 | rs78023096 | 73215056 | A | 0 | 1 |
| 5 | rs6876373 | 145376268 | G | 43 | 3 |
| 6 | rs111875628 | 32583813 | A | 169 | 107 |
| 7 | rs4143615 | 115800963 | T | 10 | 10 |
| 8 | rs146050799 | 95307788 | T | 2 | 0 |
| 8 | rs16896685 | 99155532 | A | 4 | 0 |
| 12 | rs73067158 | 18368458 | T | 1 | 0 |
| 13 | rs1428 | 92006770 | C | 1 | 0 |
| 13 | rs1572320 | 102064396 | A | 4 | 0 |
| 15 | rs62045225 | 56271008 | C | 2 | 0 |
| 18 | rs62094793 | 21402075 | G | 2 | 0 |

The chromosomal positions are based on NCBI Build 37. Chr: chromosome. The eQTL and sQTL data were queried from the GTEx portal.

**Table S7. Colocalization of rs111875628 and single-tissue eQTL results for the *HLA* class II genes.**

| **SNP** | **Tissue** | **Probe** | **Gene** | **Beta** | **SE** | ***P*** | **Han_PP_H4_** | **Tibetan_PP_H4_** |
| --- | --- | --- | --- | --- | --- | --- | --- | --- |
| rs111875628 G>A Chr6:32583813 | Whole blood | ENSG00000237541 | HLA-DQA2 | 0.528 | 0.055 | 6.17E-22 | 6.76E-69 | 1.51E-72 |
|  |  | ENSG00000229391 | HLA-DRB6 | 0.372 | 0.048 | 5.19E-15 | 2.62E-75 | 5.86E-79 |
|  |  | ENSG00000196301 | HLA-DRB9 | 0.271 | 0.047 | 1.22E-08 | 3.70E-81 | 8.27E-85 |
|  | Lung | ENSG00000237541 | HLA-DQA2 | 0.634 | 0.065 | 1.28E-22 | 2.44E-70 | 2.11E-73 |
|  |  | ENSG00000229391 | HLA-DRB6 | 0.633 | 0.060 | 2.00E-26 | 7.20E-67 | 6.22E-70 |
|  |  | ENSG00000196301 | HLA-DRB9 | 0.327 | 0.059 | 3.45E-08 | 2.10E-83 | 1.82E-86 |

SE: standard error; Han_PP_H4_: the posterior probability of H4 in the Chinese Han population; Tibetan_PP_H4_: the posterior probability of H4 in the Chinese Tibetan population.

**Table S8. The gene-set enrichment analysis for TB susceptibility in the Chinese Han population.**

| **Gene Set** | **Number of genes** | **Beta** | **Beta STD** | **SE** | ***P*** | ***P***_bon_* |
| --- | --- | --- | --- | --- | --- | --- |
| GO_bp:go_negative_regulation_of_high_voltage_gated_calcium_channel_activity | 5 | 1.667 | 0.028 | 0.366 | 2.63E-06 | **0.041** |
| GO_bp:go_lipid_hydroxylation | 6 | 1.799 | 0.033 | 0.432 | 1.59E-05 | 0.245 |
| Curated_gene_sets:reactome_runx1_regulates_transcription_of_genes_involved_in_interleukin_signaling | 5 | 1.611 | 0.027 | 0.410 | 4.38E-05 | 0.679 |
| GO_mf:go_estrogen_16_alpha_hydroxylase_activity | 5 | 1.915 | 0.032 | 0.491 | 4.82E-05 | 0.746 |
| GO_bp:go_regulation_of_b_cell_activation | 107 | 0.330 | 0.025 | 0.087 | 7.42E-05 | 1.000 |
| Curated_gene_sets:kegg_asthma | 25 | 0.718 | 0.027 | 0.191 | 8.44E-05 | 1.000 |
| GO_bp:go_regulation_of_b_cell_differentiation | 22 | 0.709 | 0.025 | 0.189 | 8.89E-05 | 1.000 |
| GO_mf:go_sulfur_compound_binding | 230 | 0.223 | 0.025 | 0.059 | 9.04E-05 | 1.000 |
| Curated_gene_sets:schaeffer_prostate_development_and_cancer_box3 | 5 | 1.267 | 0.021 | 0.340 | 9.61E-05 | 1.000 |
| GO_bp:go_regulation_of_high_voltage_gated_calcium_channel_activity | 16 | 0.757 | 0.023 | 0.205 | 1.15E-04 | 1.000 |

*, *P* value after Bonferroni correction.

**Table S9. The gene-set enrichment analysis for TB susceptibility in the Chinese Tibetan population.**

| **Gene Set** | **Number of genes** | **Beta** | **Beta STD** | **SE** | ***P*** | ***P***_bon_* |
| --- | --- | --- | --- | --- | --- | --- |
| GO_cc:go_trans_golgi_network | 220 | 0.227 | 0.025 | 0.059 | 6.20E-05 | 0.959 |
| GO_bp:go_positive_regulation_of_leukocyte_cell_cell_adhesion | 194 | 0.232 | 0.024 | 0.063 | 1.04E-04 | 1.000 |
| GO_cc:go_organelle_subcompartment | 341 | 0.174 | 0.024 | 0.047 | 1.17E-04 | 1.000 |
| GO_bp:go_positive_regulation_of_cell_cell_adhesion | 231 | 0.213 | 0.024 | 0.058 | 1.20E-04 | 1.000 |
| Curated_gene_sets:biocarta_trka_pathway | 13 | 0.808 | 0.022 | 0.221 | 1.28E-04 | 1.000 |
| GO_mf:go_epidermal_growth_factor_receptor_binding | 30 | 0.595 | 0.024 | 0.167 | 1.89E-04 | 1.000 |
| GO_bp:go_proteolysis | 1566 | 0.081 | 0.023 | 0.023 | 1.92E-04 | 1.000 |
| GO_mf:go_neurotrophin_receptor_binding | 8 | 0.980 | 0.021 | 0.278 | 2.15E-04 | 1.000 |
| Curated_gene_sets:liang_silenced_by_methylation_dn | 10 | 0.830 | 0.020 | 0.241 | 2.89E-04 | 1.000 |
| GO_bp:go_pyramidal_neuron_differentiation | 7 | 1.082 | 0.021 | 0.318 | 3.34E-04 | 1.000 |

*, *P* value after Bonferroni correction.

**Table S10. Allele frequencies for the top three SNPs associated with TB in different populations.**

| **Chr** | **SNP** | **Position** | **Ref Allele** | **Alt Allele** | [**Global**](https://www.ncbi.nlm.nih.gov/biosample/SAMN07490465) | **African** | **East Asian** | **Europe** | **South Asian** | **American** |
| --- | --- | --- | --- | --- | --- | --- | --- | --- | --- | --- |
| 6 | rs111875628 | 32583813 | G | A | 0.2540 | 0.2421 | 0.2798 | 0.2674 | 0.182 | 0.321 |
| 6 | rs114087228 | 32655830 | T | A | 0.3770 | 0.2678 | 0.4048 | 0.3290 | 0.476 | 0.474 |
| 6 | rs112925916 | 32652344 | C | T | 0.3273 | 0.2284 | 0.3403 | 0.2873 | 0.416 | 0.429 |

The chromosomal positions are based on NCBI Build 37. Chr: chromosome. The allele frequencies were obtained from the 1000 Genomes Project in different populations.

**Table S11. Power calculation for the total sample size used in the current study.**

| **Risk allele frequency** | **Genotype relative risk** | | | | | | |
| --- | --- | --- | --- | --- | --- | --- | --- |
|  | **1.100** | **1.200** | **1.300** | **1.500** | **1.900** | **2.000** | **2.200** |
| 0.010 | 0 | 0 | 0 | 0.009 | 0.299 | 0.454 | 0.744 |
| 0.030 | 0 | 0.001 | 0.020 | 0.427 | **1.000** | **1.000** | **1.000** |
| 0.050 | 0 | 0.007 | 0.131 | **0.899** | **1.000** | **1.000** | **1.000** |
| 0.100 | 0 | 0.093 | 0.694 | **1.000** | **1.000** | **1.000** | **1.000** |
| 0.200 | 0.005 | 0.522 | **0.994** | **1.000** | **1.000** | **1.000** | **1.000** |
| 0.300 | 0.015 | 0.803 | **1.000** | **1.000** | **1.000** | **1.000** | **1.000** |
| 0.350 | 0.021 | **0.866** | **1.000** | **1.000** | **1.000** | **1.000** | **1.000** |
| 0.500 | 0.031 | **0.922** | **1.000** | **1.000** | **1.000** | **1.000** | **1.000** |
| 0.750 | 0.010 | 0.689 | **0.999** | **1.000** | **1.000** | **1.000** | **1.000** |
| 0.900 | 0 | 0.091 | 0.681 | **1.000** | **1.000** | **1.000** | **1.000** |
| 0.970 | 0 | 0.001 | 0.019 | 0.404 | **0.998** | **1.000** | **1.000** |

Power was estimated for the total sample size used in the current study giving a range of Risk Allele Frequency and Genotype Relative Risk and assuming a population incidence of approximately 0.0002 and a significance level of 5×10^−8^. An adequate power of 0.850 or greater was indicated in bold.

**
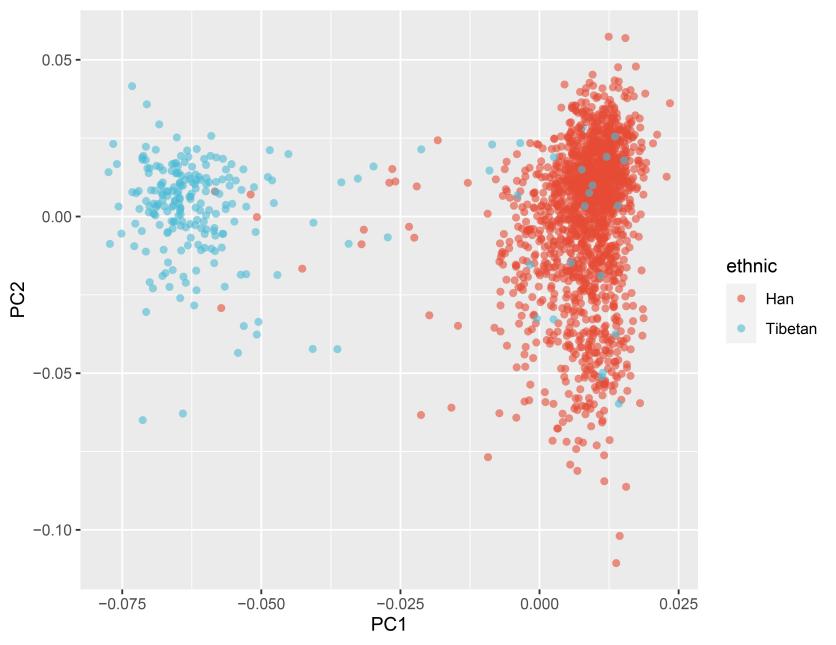
**

**Figure S1. PCA analysis of the healthy controls.** The first principal component (PC1, x-axis) was plotted against the second principal component (PC2, y-axis). The controls of the Chinese Han and Tibetan cohorts are plotted in red and blue, respectively.

**
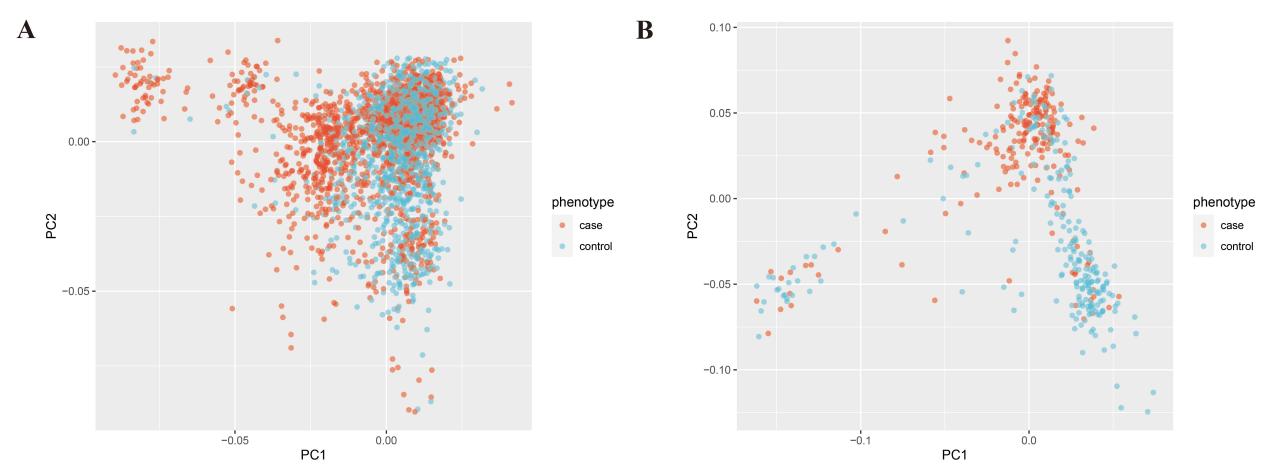
**

**Figure S2. PCA analysis of the GWAS sample. (A)** PCA analysis of the Chinese Han sample. **(B)** PCA analysis of the Chinese Tibetan sample. The first principal component (PC1, x-axis) was plotted against the second principal component (PC2, y-axis). Tuberculosis cases are plotted in red and healthy controls are plotted in blue.

**
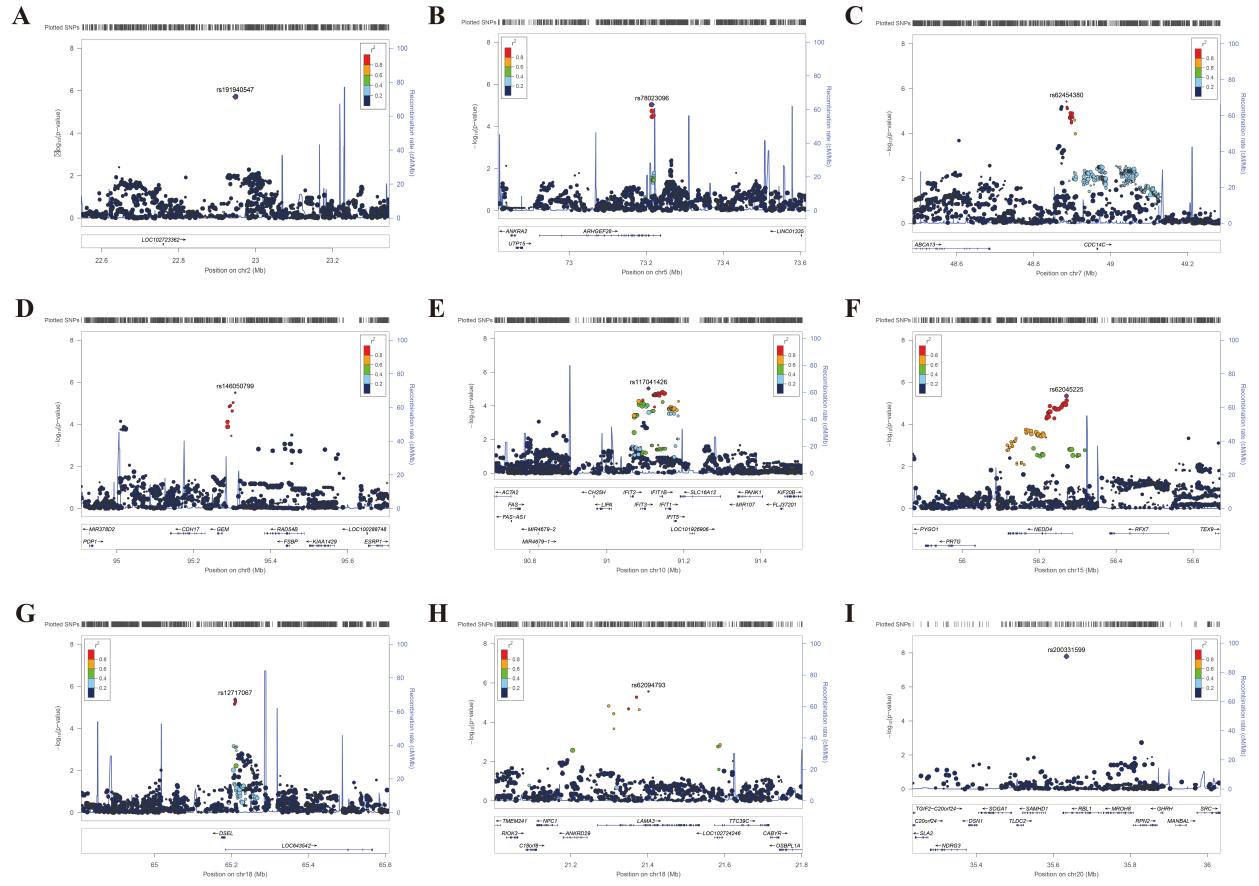
**

**Figure S3. Regional plots of suggestive loci in the Chinese Han population.** **(A-I)** Regional distribution of (A) rs191940547, (B) rs78023096, (C) rs62454380, (D) rs146050799, (E) rs117041426, (F) rs62045225, (G) rs12717067, (H) rs62094793, and (I) rs200331599 associated with TB under the additive model. The purple symbol denotes the lead SNP, and its name is shown at the top of each plot. Recombination rates are estimated from the Asian populations of the 1000 Genomes Project (Nov 2014). Gene annotations are taken from the UCSC genome browser.


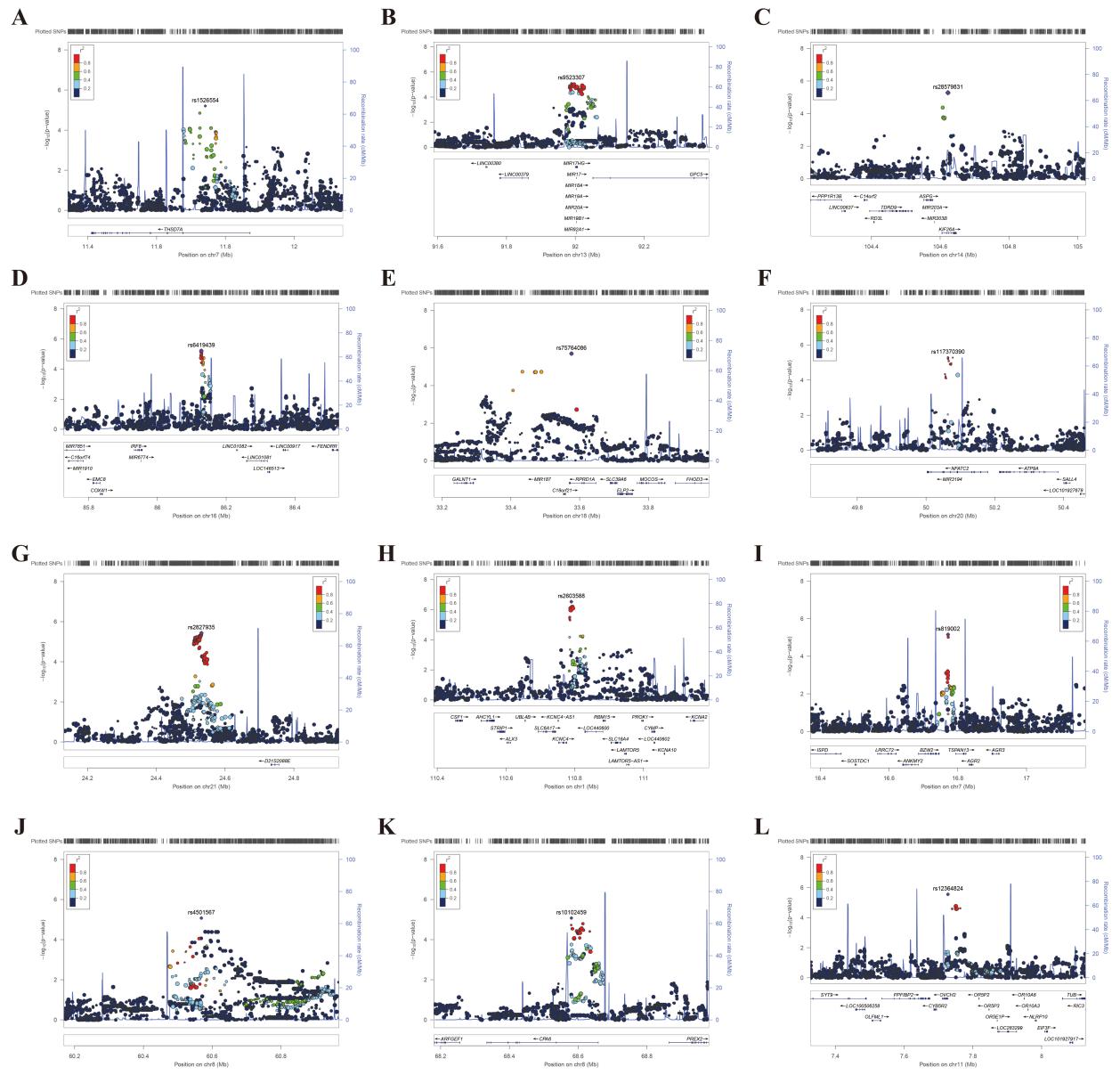


**Figure S4. Regional plots of suggestive loci in the stratification analysis of the age of diagnosis. (A-G)** Regional distribution of (A) rs1526554, (B) rs9523307, (C) rs28579831, (D) rs6419439, (E) rs75764086, (F) rs117370390, and (G) rs2827935 associated with TB in the younger group (age < 45 years) under the additive model. **(H-L)** Regional distribution of (H) rs2603588, (I) rs819002, (J) rs4501567, (K) rs10102459, and (L) rs12364824 associated with TB in the elder group (age ≥ 45 years) under the additive model. The purple symbol denotes the lead SNP, and its name is shown at the top of each plot. Recombination rates are estimated from the Asian populations of the 1000 Genomes Project (Nov 2014). Gene annotations are taken from the UCSC genome browser.

**
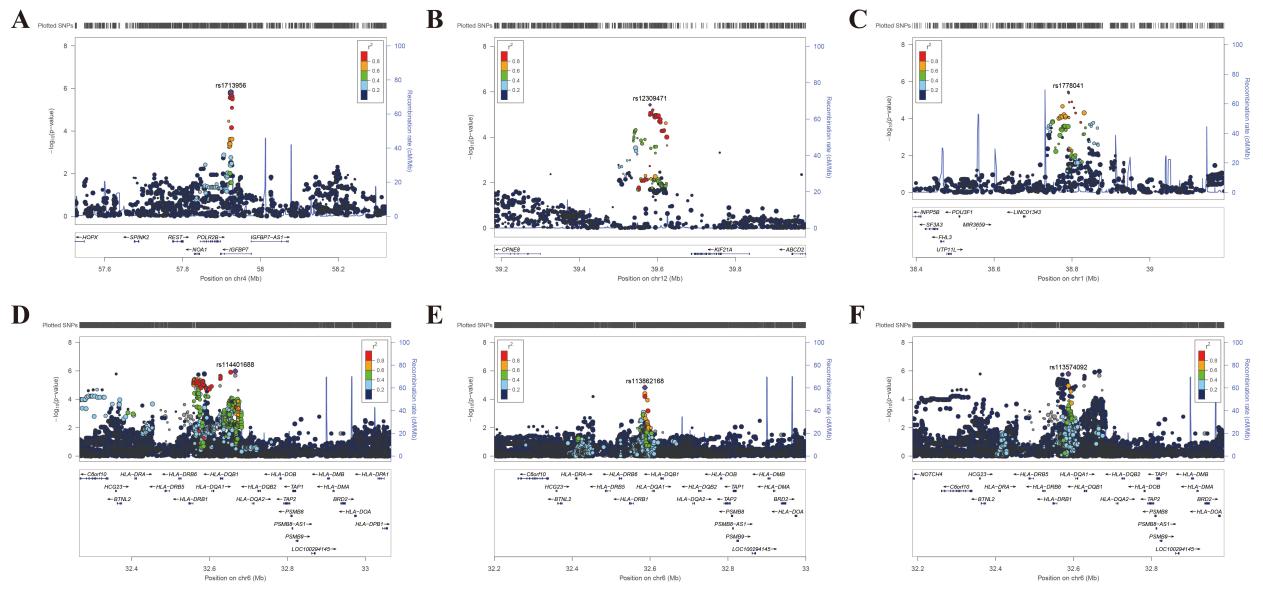
**

**Figure S5. Regional plots of suggestive loci in the stratification analysis of sex. (A-B)** Regional distribution of (A) rs1713956 and (B) rs12309471 associated with TB in the male group under the additive model. **(C-D)** Regional distribution of (C) rs1778041 and (D) rs114401688 associated with TB in the female group under the additive model. **(E)** Regional distribution of chr6. 32.2-33.0 MB of the conditional logistic regression analysis on rs114401688. **(F)** Regional distribution of rs113574092 associated with TB in the female group under the additive model. The purple symbol denotes the lead SNP, and its name is shown at the top of each plot. Recombination rates are estimated from the Asian populations of the 1000 Genomes Project (Nov 2014). Gene annotations are taken from the UCSC genome browser.

**
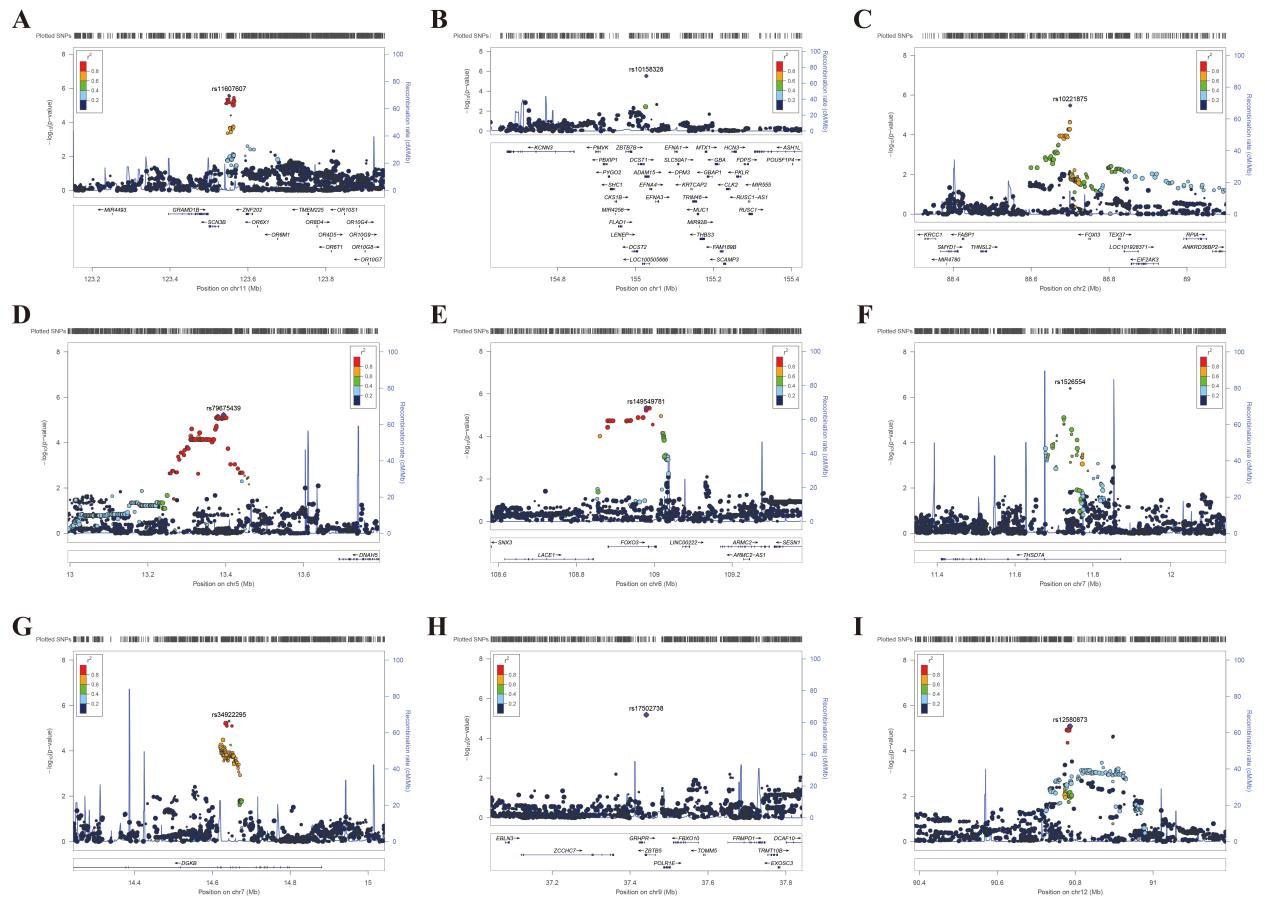
**

**Figure S6. Regional plots of suggestive loci in the stratification analysis of the clinical form of TB. (A)** Regional distribution of rs11607607 associated with PTB under the additive model. **(B-I)** Regional distribution of (B) rs10158328, (C) rs10221875, (D) rs79675439, (E) rs149549781, (F) rs1526554, (G) rs34922295, (H) rs17502738, and (I) rs12580873 associated with EPTB under the additive model. The purple symbol denotes the lead SNP, and its name is shown at the top of each plot. Recombination rates are estimated from the Asian populations of the 1000 Genomes Project (Nov 2014). Gene annotations are taken from the UCSC genome browser.


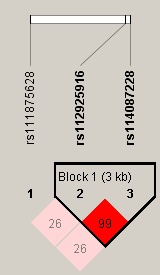


**Figure S7. The linkage disequilibrium analysis of the top three SNPs associated with TB.** The genotype data were extracted from the Chinese Han population. The linkage disequilibrium values are shown by R^2^.

**
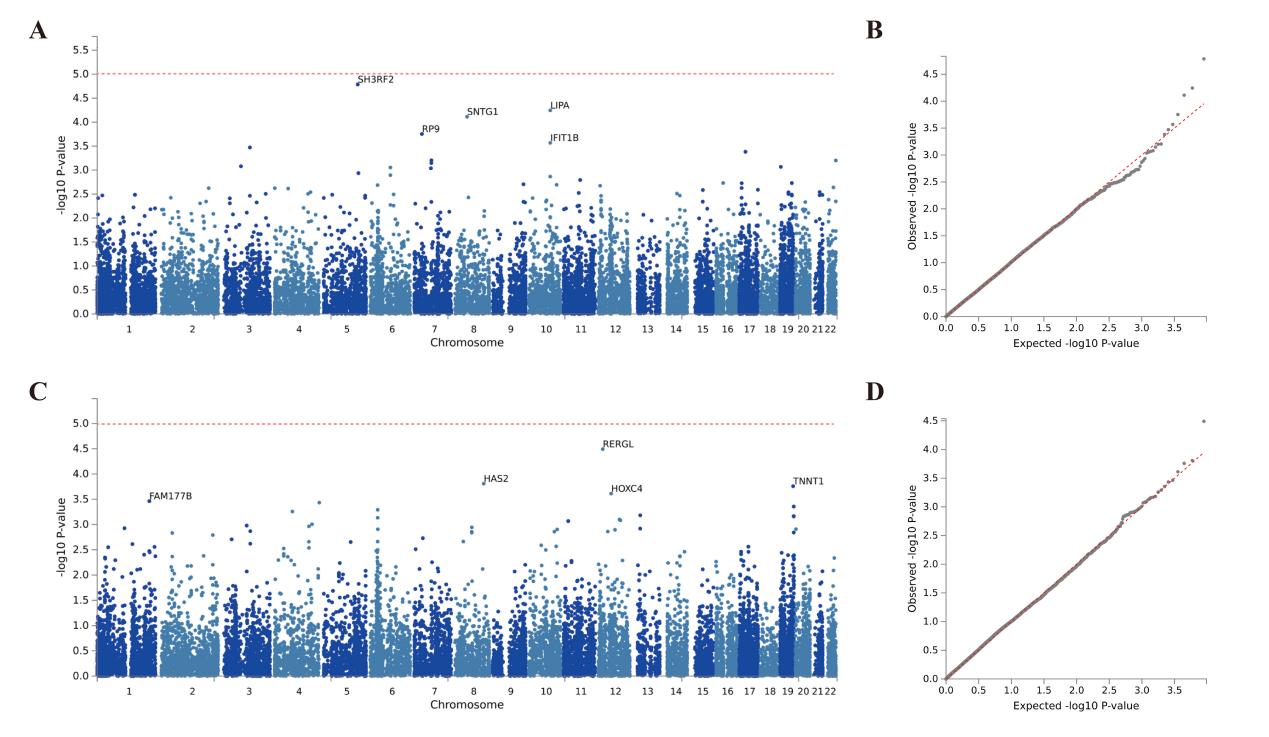
**

**Figure S8. Gene-based tests of the Chinese Han and Tibetan populations. (A)** The Manhattan plot and **(B)** Q-Q plot of the gene-based test computed by MAGMA based on the GWAS of the Chinese Han population. **(C)** The Manhattan plot and **(D)** Q-Q plot of the gene-based test computed by MAGMA based on the GWAS of the Chinese Tibetan population.
